# Investigating market-based opportunities for the provision of nutritious and safe diets to prevent childhood stunting: a UKRI-GCRF action against stunting hub protocol paper

**DOI:** 10.1136/bmjpo-2022-001671

**Published:** 2024-02-27

**Authors:** Gregory S Cooper, Hilary Davies-Kershaw, Paula Dominguez-Salas, Umi Fahmida, Babacar Faye, Elaine Ferguson, Delia Grace, Barbara N Häsler, Suneetha Kadiyala, Archana Konapur, Bharati Kulkarni, Bhagyalakshmi Chengat Prakashbabu, Indriya L Pramesthi, Dominic Rowland, Kiruthika Selvaraj, Arienta R P Sudibya, Roger C Tine, D M Dinesh Yadav, Nur L Zahra, Bhavani Shankar, Claire Heffernan

**Affiliations:** 1 Institute of Sustainable Food, Department of Geography, The University of Sheffield, Sheffield, South Yorkshire, UK; 2 Department of Population Health, London School of Hygiene and Tropical Medicine Faculty of Epidemiology and Public Health, London, UK; 3 Animal and Human Health Program, International Livestock Research Institute, Nairobi, Nairobi, Kenya; 4 Food and Markets Department, Natural Resources Institute, University of Greenwich, London, UK; 5 Southeast Asian Ministers of Education Organization Regional Centre for Food and Nutrition, Jakarta, DKI Jakarta, Indonesia; 6 Department of Nutrition, Faculty of Medicine, Universitas Indonesia, Dr. Cipto Mangunkusumo General Hospital, Jakarta, Indonesia; 7 Department of Parasitology-Mycology, Cheikh Anta Diop University of Dakar, Dakar, Senegal; 8 Department of Pathobiology and Population Sciences, The Royal Veterinary College, Hatfield, Hertfordshire, UK; 9 ICMR National Institute of Nutrition, Hyderabad, Telangana, India; 10 Center for International Forestry Research, Bogor Barat, Indonesia; 11 Centre for Environment, Development and Policy (CeDEP), SOAS, London, UK; 12 Indian Institute of Public Health, Bhubaneswar, Odisha, India; 13 London International Development Centre, London, UK

**Keywords:** Nutrition

## Abstract

**Background:**

Inadequate access to affordable, safe, desirable and convenient nutrient-dense food is one of the underlying causes of child stunting. While targeted nutrition-sensitive interventions (eg, backyard ‘nutri-gardens’) may increase dietary diversity within farming households, such interventions have limited scalability across the wider food system where markets remain underdeveloped. This research aims to develop and assess market-based interventions for key nutrient-dense foods to help improve the diets of women and children in the first 1000 days of life.

**Methods:**

Data collection uses four parallel approaches in each of the three study countries (India, Indonesia and Senegal). (1) A novel *food environment tool* will be developed to characterise the accessibility and affordability of nutrient-dense foods in the study countries. The tool will be validated through pretesting using cognitive interviewing and piloting in purposively sampled households, 10 (cognitive interviewing) and 30 (piloting) households in each country; (2) stakeholder interviews (eg, with producers, intermediaries and retailers) will be conducted to map out nutrition-sensitive entry points of key value chains (eg, animal-sourced foods), before hotspots of potential food safety hazards will be identified from food samples collected along the chains; (3) the *Optifood* and *Agrifood* tools will be used to identify foods that can address food system nutrient gaps and engage key stakeholders to prioritise market interventions to improve nutrition outcomes. *Optifood* and *Agrifood* parameters will be informed by publicly available data, plus interviews and focus groups with value chain stakeholders; (4) informed by the previous three approaches and a campaign of participatory ‘group model building’, a novel system dynamics model will evaluate the impact of alternative market-based solutions on the availability and affordability of nutrient-dense foods over time.

**Ethics and dissemination:**

The study has received ethical approval in the United Kingdom, Senegal, Indonesia and India. Dissemination comprises peer-reviewed journals, international disciplinary conferences and multistakeholder dissemination workshops.

**WHAT IS ALREADY KNOWN ON THIS TOPIC**

- Access to nutrient-dense food that is affordable, acceptable, safe and convenient is a prerequisite to counteracting food and nutrition insecurity in early life.
- In countries such as Indonesia, India and Senegal, where the ability to meet this decade’s international stunting targets remains uncertain, increasing attention is being paid to the nutrition sensitivity of market-based interventions that help to improve food storage, safety and equitable distribution.

**WHAT THIS STUDY ADDS**

- Develop and apply a novel food environment profiling tool, value chain food safety analysis and the *Optifood* and *Agrifood* models, to capture the complex socioeconomic and institutional barriers to equitable nutrition security.
- Use system-based modelling to evaluate the wider socioeconomic trade-offs and unintended consequences of nutrition-sensitive market interventions.

**HOW THIS STUDY MIGHT AFFECT RESEARCH, PRACTICE OR POLICY**

- This study will assess and recommend tailored market-based interventions within the three countries to guide decision-making around improving women and children’s year-round access to locally relevant, nutrient-dense food.

## Introduction

As of early 2020, stunting affected approximately 149 million children under the age of 5 years, with 65% of all stunted children living in low and middle-income countries (LMICs).^1 2^ The inability to achieve optimal linear growth in the early years of life is associated with a spectrum of development barriers, including the reduced ability to reach full cognitive potential, inferior rates of school achievement, economic productivity and maternal reproductive outcomes.^1 3 4^ Child stunting reaches across 8 of the 17 Sustainable Development Goals.^5^ The causes of child stunting are widely understood to be multidimensional, associated with the interactions between poverty, hunger, gender inequity, poor health, poor sanitation and poor-quality education as well as an outcome related to physically taxing work and wider structural inequalities.^6–8^

Reflecting the complex and pernicious nature of the problem, the World Health Assembly (WHA) in 2012 recognised child stunting as ‘one of the most significant impediments to human development’ and resolved to decrease the number of children under five who are stunted by 40% by 2025.^9^ However, even prior to the COVID-19 pandemic, progress around stunting reduction was acknowledged to be slowing,^2 10^ with researchers noting in 2013 that the WHA target was already on course to be missed.^11^

In an effort to evaluate the contributions of targeted interventions towards stunting reduction, the 2013 Lancet Series on Maternal and Child Nutrition found that even achieving high coverage (90%) across 10 recommended nutrition-specific interventions will likely only reduce the stunting burden in LMICs by 20%.^4 12^ Numerous studies have since promoted nutrition-sensitive strategies to provide the foundations of food and livelihood security, improved diet quality and women’s empowerment,^13–15^ on which synergistic nutrition-specific interventions can be scaled-up to help tackle stunting.^13 16 17^ In particular, increasing attention is being paid to the nutritional sensitivity of markets,^18–20^ and there is growing understanding that improved rural connectivity, cold storage and processing facilities and accessible and safe food environments may improve the equitable distribution, pricing and consumption of nutritious foods.^21–23^ Enhancing the quality of diets by harnessing such market-based solutions may be beneficial to the reduction of the risk of child stunting.

Therefore, as part of the UKRI-GCRF Action Against Stunting Hub (Jobarteh *et al*, *this issue*), a project which aims to understand the contributions of multidimensional drivers to child stunting (eg, gut health, nutrition, sanitation, education and the home environment), this particular study focuses on the role of the food system to develop and assess the potential of tailored market interventions with key nutrient-dense foods (eg, animal source foods, fruits and vegetables) to improve the diet and nutrition of pregnant and lactating women and children. Therefore, the specific objectives of the food systems component of the Action Against Stunting study are:

1. To characterise the food environments in India, Indonesia and Senegal to assess the *what*, *when*, *where* and *how* of food acquisition and consumption, with a focus on children under 2 years of age.
2. To refine and apply tools based on linear programming analyses (LPA) and multiple criteria decision analysis (MCDA) to guide decisions on how to make markets more nutrition sensitive.
3. To identify critical food safety risks and hazards in food value chains with the potential for child stunting alleviation.
4. To design nutrition-sensitive market interventions and investigate their potential to deliver nutritious foods under various future scenarios.

## Methods and analysis

### Study design

**Figure 1:**
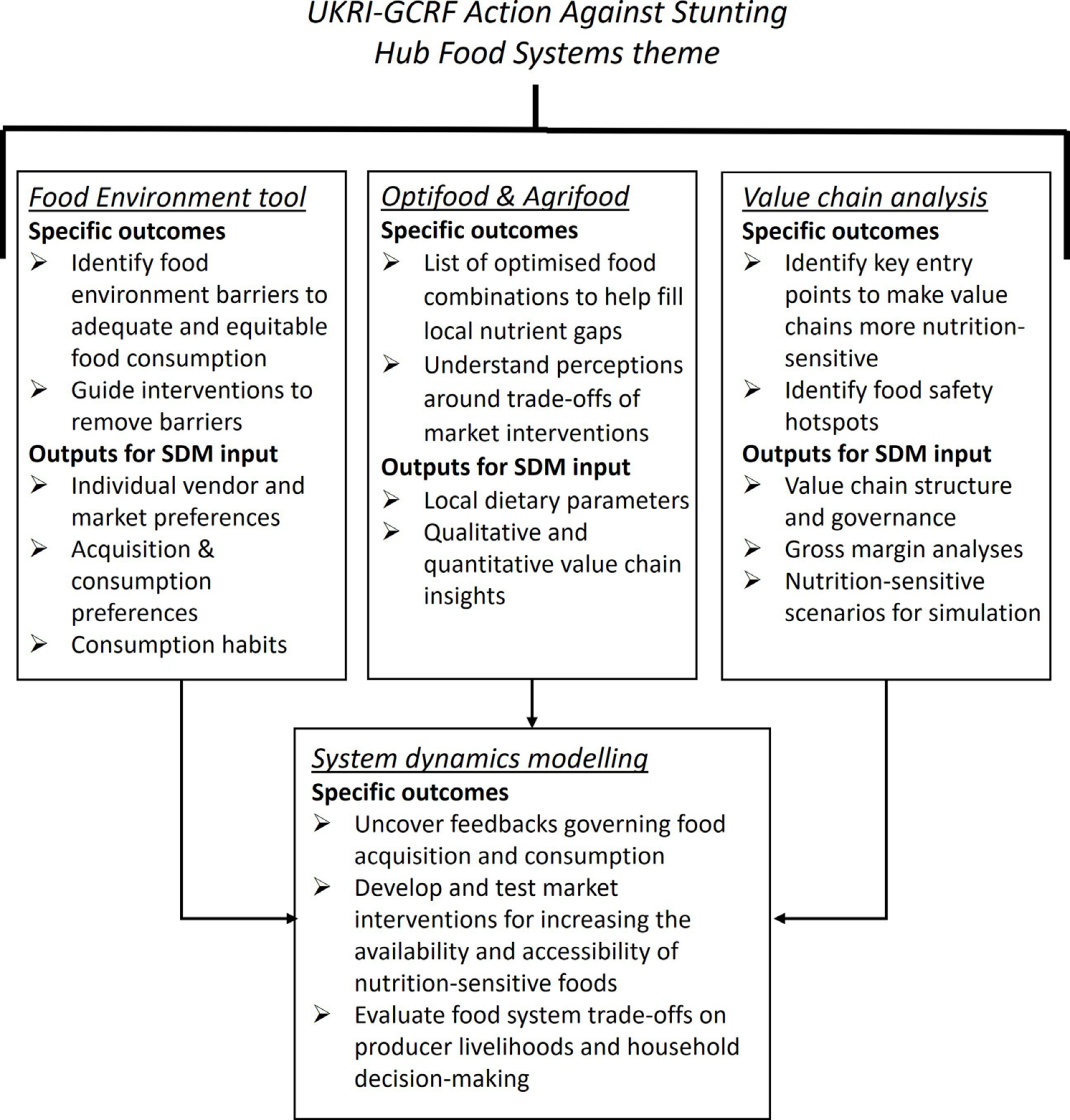
Conceptual framework linking together the sub-studies of the Food Systems theme. As denoted by the arrows, the quantitative and qualitative information generated by the three parallel data collections (Food Environment tool, Optifood and Agrifood models and value chain analysis) will directly inform the design of the system dynamics modelling activities. SDM, system dynamics modelling.

This study is part of an observational research programme into understanding the myriad of factors contributing to poor growth and development in the first 1000 days of childhood. The interdisciplinary research recruits women during pregnancy in India (Hyderabad), Indonesia (Lombok) and Senegal (Kaffrine) and follows mother–infant pairs up to 24 months after birth, with detailed investigations at predefined timepoints to support analysis into the aetiology of child stunting. This study will provide an understanding of the food environments in the study countries and its likely contribution to child stunting. Specific tools will be developed to provide in-depth analyses of the food systems, including household food environments, local markets and priority value chains, with a view to informing programmes and policies on strategies to meet the nutrient gaps in maternal and child diets. The conceptual framework—consisting of three parallel data collections, which all feed into the construction of a systems dynamic model—is depicted in figure 1.

### Characterising food environments

The food environment is the interface that mediates an individual’s food acquisition, preparation and consumption.^24^ This study will use the food environment framework developed by the Agriculture, Nutrition and Health Academy Food Environment Working Group^24^ to analyse the interactions between the two critical food environment domains (ie, external supply-side and internal demand-side factors) in the three study communities.

As detailed in the value chain mapping activity below, profiling of the supply side will include mapping distribution networks of key target nutrient-dense foods (such as dark green leafy vegetables and animal source foods such as eggs and fish) through key-informant interviews and focus group discussions. However, at present, there are no validated metrics to measure the perception of people towards their local food environments in LMICs.^23 24^ As a result, we will develop a standardised Food Environment Experience tool to measure the perceived affordability, safety (physical and food safety) and physical and financial accessibility of food environments across the three study locations (see table 1 for a full list of food environment concepts captured by the tool).

**Table 1:** Descriptions of the food environment concepts captured in the Food Environment Experience tool, built on the food environment dimensions and domains detailed in Turner *et al* 24

Dimension	Domain	Concept in the Food Environment Experience tool	Sample question from Food Environment Experience tool
Activity space	Personal and external	The places that household members visit as a part of their daily routine	*In the last 30 days, what are all the places from which you or other household members have visited, as a part of your daily routine, to get food for household members to consume?*
Availability	External	Food product was observed to be present by the respondent	*In the last 30 days, in the places you visit as a part of your daily routine, how often were DGLVs available?*
Vendor properties	External	Potential to acquire food product on credit	*If you did try to get DGLVs on credit with written agreement during the last 30 days, how often was it possible to do so successfully?*
	External	Food quality, comprised of food safety and hygiene	*Thinking of all the places where DGLVs were available in the last 30 days, how often did you see DGLVs that you would consider hygienic (regardless of whether you got them or not)?*
Marketing and regulation	External	Promotional information (eg, nutritional messages, adverts, special offers)	*During the last 30 days, how often did you read, hear, or watch advertisements to buy DGLVs?*
Physical accessibility	Personal and external	Experience of physical safety and security	*In the last 30 days, how often did you find dark DGLVs in areas that you would consider safe to go to?*
	Personal and external	Ease of physical access	*In the last 30 days, how often did you find DGLVs in places that are easy to go to?*
Affordability	Personal and external	Food product priced at a cost that the household could spend	*During the last 30 days, in the places you have visited as a part of your daily routine, how often did you find DGLVs that were priced at a cost that your household could spend?*
Convenience	Personal	Sufficient time to acquire, prepare and cook food product	*In your opinion, were there any times in the last 30 days when you or your household members did not have sufficient time to get DGLVs for household consumption? If yes, how often did you NOT have sufficient time to get DGLVs?*
Desirability	Personal	Tastes, taboos and cultural compatibility, health attitudes and knowledge	*Do people who are important to you (eg, family/friend) like the taste of DGLVs? If yes, how strongly do they feel?*

DGLVs, dark green leafy vegetables.

The tool will be pretested in two stages. First, cognitive interviewing^25^ will be used to explore the extent to which respondents understand the meaning of key concepts (eg, *affordability, food acquisition* and *activity space*). Series of semistructured interviews with Likert scales and local language translations (ie, Telugu, French and Bahasa Indonesia) will be conducted. The cognitive questionnaire will first be piloted in a purposively sampled set of respondents not enrolled in the interdisciplinary observational study. In each country, two trained staff will conduct 10 interviews within different demographic (urban and rural) and socioeconomic conditions. Transcripts of the interviews will be analysed across two rounds using standard thematic analysis to guide improvements to the wording and framing of questions.^26 27^

Second, the revised questionnaire will be piloted within a purposively sampled set of 30 mothers and children under 2 years of age in each of the study communities. This second testing will again be conducted in mother–infant pairs not included in the observational study. The data generated from the two validation studies will inform the development of a novel Food Environment Experience tool prototype.

The food environment questionnaire will then be administered to mothers enrolled in the observational study at 9 and 12 months postpartum. With the recruitment of cohort households happening at over the course of 1 year, staggering the data collection across successive months will provide insights into how the availability and affordability of nutrient-dense foods vary seasonally (ie, 12 successive months of data from May 2022 to April 2023 for dark green leafy vegetables in Hyderabad). In turn, this questionnaire will uncover insights into why people prioritise particular food environment domains when making food decisions as well as helping to inform interventions that have the potential to overcome community-level food accessibility barriers.

### Optifood and Agrifood modelling to improve food systems

The state-of-the-art Optifood and Agrifood^28^ tools will be used to: (1) identify foods that can address the nutrient gaps in an individual’s diet, (2) guide market-based intervention development, (3) identify priority food value chains for assessment and (4) identify model parameters for the systems dynamic modelling. The model parameters which will be used in the Optifood and Agrifood tools will be defined using primary data (ie, dietary intakes) from the observational study, stakeholder workshops, the novel food environment tool and secondary data from Stunting Hub household dietary surveys conducted in the countries. All dietary intakes in the Action Against Stunting Hub (AASH) observation study will be collected using the four-pass 24-hour (MP24HR) dietary recall method.^29^ Maternal intakes will be collected during the second and third trimesters in East Lombok, Indonesia and Kaffrine, Senegal and during the third trimester in Hyderabad, India. Infant dietary intake will be collected at 6, 9, 12, 18 and 24 months after birth. A second MP24HR will be collected from a 10% random subsample from each country site at each time point to reduce random measurement error.^30^

The analyses will be conducted using LPA in *Optifood* and MCDA in *Agrifood*. The dietary data obtained from the observational study and dietary surveys (secondary data) will be used in *Optifood* to generate a list of foods and subgroups of foods consumed in the communities to identify gaps in the nutrient adequacy of maternal and child diets. As a scenario building decision-making tool, *Agrifood* will use these lists, as well as multiple criteria, for decision-making on how to improve the accessibility, availability or affordability of identified foods/food subgroups via market-based interventions. This will involve conducting workshops with different stakeholders, including community members, value chain stakeholders (eg, vendors, retailers, traders) and individuals within decision-making organisations (eg, consumer rights organisations and producer associations). Discussion topics will include how decisions are prioritised in the food system and the value stakeholders place on these criteria. The results of the *Agrifood* analyses will be shared, in another workshop, with these stakeholders to generate discussions related to trade-offs of alternative choices from different stakeholder group perspectives.

### Food safety hazards and risks in value chains

Food safety hazards and risks will be assessed along selected value chains for foods with potential to reduce stunting. Commodity selection will be based on the following criteria: (a) ability to address nutrition gaps, (b) acceptability to consumers, (c) acceptability to key value chain stakeholders and (d) amenability to solutions for reduced risk of food-born illnesses.

The observational study will investigate the priority nutrient-dense foods in the study countries. The value chain analysis will be linked to the food environment research to further investigate the sources of nutrient-dense foods (ie, where people are getting the foods) as well as modes of transportation, storage, processing and consumption. The sources of the food including the point-of-purchase information will be used to link households to a particular food environment. A combination of qualitative and quantitative methodologies, including biological sampling and testing^31 32^ (specifically for pathogens such as E.coli, Salmonella, Shigella, and Campylobacter), will be employed in the value chains for identification of food safety hazards and risks.

It is expected that the priority foods will be delivered by numerous value chains to vulnerable consumers. Thus, the selection of specific value chains to focus on will be based on their contribution to the diets of the target populations, known food safety risks as established in existing literature, and its relevance to stunting in the country context. Based on existing evidence on local diets and food safety risks, likely candidates are fish, milk and eggs.

Processes, people, products, animals, flows of information and money, practices, regulations and governance will be assessed for up to five selected value chains per product. This will provide a detailed chain mapping, characterisation of attributes and risks, seasonality, food safety perceptions and behaviours, formal and informal rules and governing structures.^31 32^ Data will be collected through interviews, direct observation, focus group discussions, surveys and geographical information from key informants, including primary producers, commission agents and retailers. High-level representatives will be also interviewed, such as those from relevant Ministries or the Food Safety authority. Food samples of priority value chains at critical chain nodes will be taken and analysed for biological hazards, namely, at the following locations: retail and wholesale markets, transportation, bulking point (eg, milk collection centre), harvest, production and preharvest inputs. Finally, we will use the information gathered to identify leverage points for cost-effective interventions, to make these foods safer and more available.

### Modelling nutrition-sensitive interventions

The resource intensity of standard experimental approaches is a major constraint in estimating the implications of market interventions on child stunting prevention. Furthermore, such experimental designs have limited ability to generate policy-relevant information at scale,^33^ including the emergence of trade-offs over time. System dynamics modelling (SDM) is a microsimulation technique which represents system stocks, flows and feedbacks in non-linear differential equations.^21^ Using data collected by the *Agrifood* tool, and food environment and value chain assessments, SDM will be used to conduct ‘what-if’ scenario analysis to identify potential interventions to improve the availability and affordability of nutrient-dense foods in markets over time.

SDM will also use data collected from a campaign of participatory ‘group model building’ (GMB) in each of the three study sites,^34^ which will be focused on discovering the feedbacks underlying household decision-making around food acquisition and consumption. As previously conducted in Bihar and Myanmar,^34^ local stakeholders will be first introduced to systems concepts, before participatory discussions on the most important feedbacks. A third session will focus on generating quantitative data to numerically parameterise the models, and then a final session to provide an opportunity to cooperatively evaluate the draft model. The LayerStack offline Geographical Information System will be used in the participatory discussions to enable stakeholders to map and visualise system components across space and time.^34^

The sessions will involve up to 15 local stakeholders involved in retail and purchase of nutrient-dense foods, 2–3 facilitators-cum-translators and a specialist modeller to code insights into the modelling software (ie, STELLA–ISEE systems)^34–36^ per country. Discussions will take place in interview and focus group formats, whereby the modeller and facilitators guide the discussions through a series of preplanned topics on the major stocks, flows and feedbacks governing food system dynamics. Purposeful sampling will be used to select participants who are able to participate up to 3 hours per week, for 4 weeks; where socioculturally appropriate, separate sessions will be held for female and male participants.

The formal model will be quantitatively evaluated against secondary time-series data and qualitatively with a reference group of 3–4 local experts.^34^ Future scenarios will then explore the impacts of nutrition-sensitive interventions (eg, healthy food promotions or cold storage construction) on the evolution of child stunting-sensitive outcomes, including the seasonal availability of our target food item of interest (eg, dark green leafy vegetables) and its distribution and consumption within households.

### Data management and analysis

Data will be collected following standardised protocols for value chain mapping, focus group discussions, key-informant interviews and GMB. Interview guides will be developed in English, translated into local languages and pre-tested before data collection. All the interviews and focus groups will be conducted by trained local research assistants fluent in the local language. Informed consent will be obtained from participants before collecting data and audio-recordings. All participants will be assured of the confidentiality of the questionnaire responses and food sampling data. All primary data will be stored on password-protected laptops and desktops in line with central Action Against Stunting Hub protocols.

For the MCDA and LPA, the *Optifood* and *Agrifood* software programmes will be used.^28^ Qualitative data from value chain interviews and focus groups will be analysed thematically using coding software (eg, Nvivo). Quantitative analysis of the food environment tool, value chain assessments and SDM outputs will be conducted using appropriate statistical software (eg, STELLA-ISEE Systems and R statistical software). Analysis of the Food Environment Experience tool will focus on three dimensions. First, to both compare between food products and locations at an aggregated level, summary statistics such as weighted averages (ie, weighted by the number of households which visited each source to obtain food for household consumption) and measures of variance will describe the overall levels of food availability, affordability and experiences of individual food environment dimensions (table 1). Second, standard correlation analysis (eg, Peason’s r) and linear regression modelling will explore whether potential links exist between the food environment experiences of individual households and their food acquisition behaviours over the last 30 days. Third, linear dimensionality reduction techniques, such as principal component analysis and factor analysis, will explore the importance of individual subdomains towards the overall food environment experience dimensions (eg, convenience of food procurement vs convenience of food preparation).

In turn, both the validation and simulation of the SDM model will use standard Monte Carlo techniques to (a) understand the extent to which the model can replicate historical patterns of system behaviour (eg, seasonal dynamics in nutrient-dense food availability)^37^ and (b) explore the extent to which uncertainties in model design and parameterisation influence the reliability of model projections.^36^

### Patient and public involvement

Study participants (mothers) and public members (stakeholders and experts) will be involved in value chain interviews, food environment surveys and GMB. Key local decision-makers will be involved in research dissemination. Partners at the National Institute of Nutrition (NIN-India), Southeast Asian Ministers of Education Organization Regional Center for Food and Nutrition (SEAMEO-RECFON-Indonesia) and University of Cheikh Anta DIOP (Senegal) will facilitate these activities.

## Ethics and dissemination

Ethical approval of the study was granted by the institutional ethics committee of the London School of Hygiene and Tropical Medicine (17915/RR/17513), by the Social Science Research Ethical Review Board at the Royal Veterinary College (URN SR2020-0197) and by the International Livestock Research Institute Institutional Research Ethics Committee (ILRI-IREC2020-33). Moreover, in-country ethics approvals were granted by the following bodies: National Institute of Nutrition (ICMR), Ministry of Health and Family Welfare, Government of India (CR/04/I/2021); Health Research Ethics Committee, University of Indonesia and Cipto Mangunkusumo Hospital (KET-887/UN2.F1/ETIK/PPM.00.02/2019); Comité National d'Ethique pour la Recherche en Santé, Senegal (Protocole SEN19/78). Ethical approval for the Food Systems theme was granted by the Ethics Committee of the London School of Hygiene and Tropical Medicine (17919).

Initial findings will be disseminated via seminars, public engagement events and relevant international conferences. Findings of the study will be published in peer-reviewed journals. Where possible, the publications will encourage authorship of early-career researchers in the research. Finally, the data sets and models generated from the study will be deposited in a public data repository.

## Supplementary Material

Author's manuscript
