# Supplementary material for: Investigating market-based opportunities for the provision of nutritious and safe diets to prevent childhood stunting: a UKRI-GCRF action against stunting hub protocol paper
Source: BMJ Paediatr Open. 2024 Feb 27;8(Suppl 1):e001671. doi: 10.1136/bmjpo-2022-001671 (PMC10900371; doi:10.1136/bmjpo-2022-001671)
Supplement: Author's manuscript [file bmjpo-2022-001671.draft_revisions.pdf]

BMJ Paediatrics Open

BMJ Paediatrics Open is committed to open peer review. As part of this commitment we make the peer review history of every article we publish publicly available.

When an article is published we post the peer reviewers' comments and the authors' responses online. We also post the versions of the paper that were used during peer review. These are the versions that the peer review comments apply to.

The versions of the paper that follow are the versions that were submitted during the peer review process. They are not the versions of record or the final published versions. They should not be cited or distributed as the published version of this manuscript.

BMJ Paediatrics Open is an open access journal and the full, final, typeset and author-corrected version of record of the manuscript is available on our site with no access controls, subscription charges or pay-per-view fees (http://bmjpaedsopen.bmj.com).

If you have any questions on BMJ Paediatrics Open's open peer review process please email info.bmjpo@bmj.com

BMJ Paediatrics Open

Investigating market-based opportunities for the provision of nutritious and safe diets to prevent childhood stunting: a UKRI-GCRF Action Against Stunting Hub protocol paper

Journal:	*BMJ Paediatrics Open*
Manuscript ID	bmjpo-2022-001671
Article Type:	Protocol
Date Submitted by the Author:	12-Sep-2022
Complete List of Authors:	Cooper, Gregory; The University of Sheffield, Institute of Sustainable Food and Department of Geography<br>Davies-Kershaw, Hilary; London School of Hygiene and Tropical Medicine Faculty of Epidemiology and Public Health, Department of Population Health<br>Dominguez-Salas, Paula; International Livestock Research Institute; University of Greenwich, Natural Resources Institute<br>Fahmida, Umi; Southeast Asian Ministers of Education Organization Regional Centre for Food and Nutrition<br>Faye, Babacar; Cheikh Anta Diop University of Dakar, Department of Parasitology-Mycology<br>Ferguson, Elaine; London School of Hygiene and Tropical Medicine Faculty of Epidemiology and Public Health<br>Grace, Delia; International Livestock Research Institute<br>Häsler, Barbara N; The Royal Veterinary College Department of Pathobiology and Population Sciences<br>Kadiyala, Suneetha; London School of Hygiene and Tropical Medicine Faculty of Epidemiology and Population Health, Department of Population Health<br>Konapur, Archana; ICMR National Institute of Nutrition<br>Kulkarni, Bharati; ICMR National Institute of Nutrition<br>Prakashbabu, Bhagyalakshmi ; The Royal Veterinary College Department of Pathobiology and Population Sciences<br>Pramesthi, Indriya ; Southeast Asian Ministers of Education Organization Regional Centre for Food and Nutrition<br>Rowland, Dominic; Center for International Forestry Research; SOAS, Centre for Environment, Development and Policy (CeDEP)<br>Selvaraj, Kiruthika; ICMR National Institute of Nutrition<br>Shankar, Bhavani; The University of Sheffield, Institute of Sustainable Food and Department of Geography<br>Sudibya, Arienta R.P.; Southeast Asian Ministers of Education Organization Regional Centre for Food and Nutrition<br>Tine, Roger ; Cheikh Anta Diop University of Dakar, Department of Parasitology-Mycology<br>Yadav, Dinesh; ICMR National Institute of Nutrition<br>Zahra, Nur ; Southeast Asian Ministers of Education Organization Regional Centre for Food and Nutrition<br>Heffernan, Claire; The Royal Veterinary College Department of

	Pathobiology and Population Sciences; London International Development Centre
Keywords:	Nutrition

SCHOLARONE™ Manuscripts

I, the Submitting Author has the right to grant and does grant on behalf of all authors of the Work (as defined in the below author licence), an exclusive licence and/or a non-exclusive licence for contributions from authors who are: i) UK Crown employees; ii) where BMJ has agreed a CC-BY licence shall apply, and/or iii) in accordance with the terms applicable for US Federal Government officers or employees acting as part of their official duties; on a worldwide, perpetual, irrevocable, royalty-free basis to BMJ Publishing Group Ltd ("BMJ") its licensees and where the relevant Journal is co-owned by BMJ to the co-owners of the Journal, to publish the Work in this journal and any other BMJ products and to exploit all rights, as set out in our licence.

The Submitting Author accepts and understands that any supply made under these terms is made by BMJ to the Submitting Author unless you are acting as an employee on behalf of your employer or a postgraduate student of an affiliated institution which is paying any applicable article publishing charge ("APC") for Open Access articles. Where the Submitting Author wishes to make the Work available on an Open Access basis (and intends to pay the relevant APC), the terms of reuse of such Open Access shall be governed by a Creative Commons licence – details of these licences and which Creative Commons licence will apply to this Work are set out in our licence referred to above.

Other than as permitted in any relevant BMJ Author's Self Archiving Policies, I confirm this Work has not been accepted for publication elsewhere, is not being considered for publication elsewhere and does not duplicate material already published. I confirm all authors consent to publication of this Work and authorise the granting of this licence.

Confidential: For Review Only

Dr Gregory S. Cooper
Institute for Sustainable Food
University of Sheffield
Sheffield, UK
S10 2TN

12th September 2022

Dear Editor-in-Chief,

Submitted as part of the ‘UKRI-GCRF Action Against Stunting’ supplement, we wish the editors to consider our manuscript titled “Investigating market-based opportunities for the provision of nutritious and safe diets to prevent childhood stunting: a UKRI-GCRF Action Against Stunting Hub protocol paper” for publication in *BMJ Paediatrics Open*.

Over the last two decades, efforts to improve food and nutrition security in low and middle-income countries have focused on policies seeking to diversify agricultural production and supplement household food baskets through public distribution systems. Building on both the successes and drawbacks of these first generation ‘agri-nutrition convergence’ approaches, food markets and their associated value chains are increasingly being seen as potentially powerful leverage points to scale-up access to nutrient-dense foods, for example, inclusive of landless communities who may not have primary means of agricultural production.

Therefore, acknowledging that access to affordable, safe, and culturally acceptable nutrient-dense foods (e.g., fruits and vegetables and animal-sourced foods) is a key facet of the ‘whole child approach’ to tackling childhood stunting, we detail the rationale, aims and four interlinked methodological approaches of the Food Systems theme in this protocol. First, we detail the design and pre-testing strategy of a novel ‘Food Environment Experience’ survey, which aims to characterise the perceptions that study cohort mothers hold towards the safety, accessibility, and affordability of the local retail food environment. Second, we detail the parameterisation of the *Optifood* and *Agrifood* models, which will be used to identify foods that can address the nutrient gaps in an individual’s diet. Third, we describe our value chain and food safety sampling approaches, which aim to identify where and why food safety hazards are introduced into the value chains of nutrient-dense foods. Lastly, informed by the previous three stages, we describe our strategy to design quantitative system dynamics models used to conduct ‘what-if’ scenario analysis to identify potential interventions to improve the availability and affordability of nutrient-dense foods in markets over time.

We have chosen to submit to *BMJ Paediatrics Open* as we believe our contribution cuts across multiple ongoing debates that are interesting to the journal’s audience, including the need to tackle childhood stunting through an integrated ‘whole child approach’, with contributions from public health, public policy, economics, and nutrition. We can confirm that this work has not been published and is not being considered elsewhere. No potential conflicts of interest are reported. We thank you for considering our paper for publication in *BMJ Paediatrics Open* and look forward to your response.

Yours sincerely,

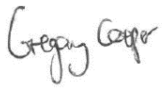

Dr. Gregory S. Cooper (corresponding author)
University of Sheffield
Sheffield, United Kingdom

Confidential: For Review Only

Investigating market-based opportunities for the provision of nutritious and safe diets to prevent childhood stunting: a UKRI-GCRF Action Against Stunting Hub protocol paper

Gregory S Cooper[a]., Hilary Davies-Kershaw[b]., Paula Dominguez-Salas[c,d]., Umi Fahmida[e]., Babacar Faye[f]., Elaine L Ferguson[b]., Delia Grace[c]., Barbara N Häsler[g]., Suneetha Kadiyala[b]., Archana Konapur[h]., Bharati Kulkarni[h]., Bhagyalakshmi C Prakashbabu[g]., Indriya L Pramesthi[e]., Dominic Rowland[i,j]., Kiruthika Selvaraj[h]., Bhavani Shankar[a]., Arienta R P Sudibya[e]., Roger C Tine[f]., Dinesh Yadav[h]., Nur L Zahra[e]., Claire Heffernan[g,k]

Affiliations

[a] Institute for Sustainable Food and Department of Geography, University of Sheffield, Sheffield, United Kingdom

[b] Department of Population Health, London School of Hygiene and Tropical Medicine, London, United Kingdom

[c] International Livestock Research Institute (ILRI), Nairobi, Kenya

[d] Natural Resources Institute, University of Greenwich, London, United Kingdom

[e] Southeast Asian Ministers of Education Organization Regional Center for Food and Nutrition (SEAMEO-RECFON)-Pusat Kajian Gizi Regional, Universitas Indonesia, Jakarta, Indonesia

[f] Department of Parasitology-Mycology, University of Cheikh Anta DIOP, Dakar, Senegal

[g] Department of Pathobiology and Population Sciences, Royal Veterinary College (RVC), Hatfield, United Kingdom

[h] ICMR- National Institute of Nutrition (ICMR), Hyderabad, Telangana, India

[i] Center for International Forestry Research, Bogor, Indonesia

[j] Centre for Development, Environment and Policy (CeDEP), SOAS University of London, London, United Kingdom

[k] London International Development Centre (LIDC), London, United Kingdom

Corresponding author: Gregory S Cooper, Institute for Sustainable Food and Department of Geography, University of Sheffield, Sheffield, United Kingdom, g.s.cooper@sheffield.ac.uk

ABSTRACT

Background

Inadequate access to affordable, safe, desirable and convenient nutrient-dense food is one of the underlying causes of child stunting. Whilst targeted nutrition-sensitive interventions (e.g., backyard 'nutri-gardens') may increase dietary diversity within farming households, such interventions have limited scalability across the wider food system where markets remain underdeveloped. This research aims to develop and assess market-based interventions for key nutrient-dense foods to help improve the diets of women and children in the first 1000 days of life.

Methods

Data collection uses four approaches in each of the three study countries (India, Indonesia and Senegal). (1) A novel *food environment tool* will be developed to characterise the accessibility and affordability of nutrient-dense foods in the study countries. The tool will be validated through pre-testing using cognitive interviewing and piloting in purposively sampled households, 10 (cognitive interviewing) and 30 (piloting) households in each country. (2) Stakeholder interviews (e.g., with producers, intermediaries and retailers) will be conducted to map out nutrition-sensitive entry points of key value chains (e.g., animal-sourced foods), before hotspots of potential food safety hazards will be identified from food samples collected along the chains. (3) The *Optifood* and *Agrifood* tools will be used to identify foods that can address food system nutrient gaps and engage key stakeholders to prioritise market interventions to improve nutrition outcomes. *Optifood* and *Agrifood* parameters will be informed by publicly available data, plus interviews and focus groups with value chain stakeholders. (4) Informed by the previous three approaches and participatory 'group model building' sessions, a novel system dynamics model will evaluate the impact of alternative market-based solutions on the availability and affordability of nutrient-dense foods over time.

Ethics and dissemination

The study has received ethical approval in the United Kingdom, Senegal, Indonesia and India. Dissemination comprises peer-reviewed journals, international disciplinary conferences, and multi-stakeholder dissemination workshops.

Confidential: For Review Only

What is already known on this topic?

- Access to nutrient-dense food that is affordable, acceptable, safe and convenient is a prerequisite to counteracting the multidimensional causes of stunting in early life.
- Despite gradual gains over the past decade, the ability of Indonesia, Senegal and India to meet international stunting reduction targets by 2025 remains uncertain.
- There is increasing evidence that market-based interventions that improve food storage, safety and distribution can work synergistically with supply-side interventions to improve equitable nutrition access.

What this study hopes to add?

- To develop, assess and recommend tailored market-based interventions to improve women and children's year-round access to locally relevant, nutrient-dense food.
- To develop a food environment profiling tool, value chains food safety analysis, and the application of *Optifood* and *Agrifood* to inform intervention selection.
- To inform policy-making by exploring the wider socioeconomic trade-offs and unintended consequences of nutrition-sensitive market interventions through system dynamics modelling.

Confidential: For Review Only

INTRODUCTION

As of early-2020, stunting affected approximately 149-million children under the age of five years, with 65% of all stunted children living in low- and middle-income countries (LMICs).[1,2] The inability to achieve optimal linear growth in the early years of life is associated with a spectrum of development barriers, including the reduced ability to reach full cognitive potential, inferior rates of school achievement, economic productivity and maternal reproductive outcomes.[1,3,4] Child stunting reaches across eight of the 17 Sustainable Development Goals (SDGs),[5] being the pernicious face of poverty, hunger, gender inequity, poor health, poor sanitation and poor quality education, as well as an outcome related to physically taxing work and wider structural inequalities.[6–8]

Reflecting the multidimensional nature of the problem, the World Health Assembly (WHA) in 2012 recognized child stunting as ‘one of the most significant impediments to human development’ and resolved to decrease the number of children under five who are stunted by 40% by 2025.[9] However, even prior to the COVID-19 pandemic, progress around stunting reduction was acknowledged to be slowing,[2,10] with researchers noting in 2013 that the WHA target was already on course to be missed.[11]

In an effort to evaluate the contributions of targeted interventions towards stunting reduction, the 2013 Lancet Series on Maternal and Child Nutrition found that even achieving high coverage (90%) across 10 recommended nutrition-specific interventions will likely only reduce the stunting burden in LMICs by 20%.[4,12] Numerous studies have since promoted nutrition-sensitive strategies to provide the foundations of food and livelihood security, improved diet quality and women’s empowerment,[13–15] upon which synergistic nutrition-specific interventions can be scaled-up to tackle stunting.[13,16,17] In particular, increasing attention is being paid to the nutritional sensitivity of markets,[18–20] and there is growing understanding that improved rural connectivity, cold storage and processing facilities, and accessible and safe food environments may improve the equitable distribution, pricing and consumption of nutritious foods.[21–23] Enhancing the quality of diets by harnessing such market-based solutions may be beneficial to the reduction of the risk of child stunting.

The aim of this study is to develop and assess the potential of tailored market interventions with key nutrient-dense foods (e.g., animal source foods, fruits and vegetables) to improve the diet and nutrition of pregnant and lactating women and children. The specific objectives of the study are:

1. To characterise the food environments in India, Indonesia and Senegal to assess the *what*, *when*, *where* and *how* of food acquisition and consumption, with a focus on children under two years of age.
2. To refine and apply tools based on linear programming analyses (LPA) and multiple criteria decision analysis (MCDA) to guide decisions on how to make markets more nutrition-sensitive.
3. To identify critical food safety risks and hazards in food value chains with the potential for child stunting alleviation.
4. To design nutrition-sensitive market interventions and investigate their potential to deliver nutritious foods under various future scenarios.

Confidential: For Review Only

METHODS AND ANALYSIS

Study design

This study is part of an observational research programme into understanding the myriad of factors contributing to poor growth and development in the first 1000 days of childhood. The interdisciplinary research recruits women during pregnancy in India (Hyderabad), Indonesia (Lombok) and Senegal (Kaffrine) and follows mother-infant pairs up to 24 months after birth, with detailed investigations at predefined timepoints to support analysis into the aetiology of child stunting. This study will provide an understanding of the food environments in the study countries and its likely contribution to child stunting. Specific tools will be developed to provide in-depth analyses of the food systems including household food environments, local markets, and priority value chains, with a view to informing programmes and policies on strategies to meet the nutrient gaps in maternal and child diets. The conceptual framework of the study including details of the tools for assessing the food system is depicted in Figure 1.

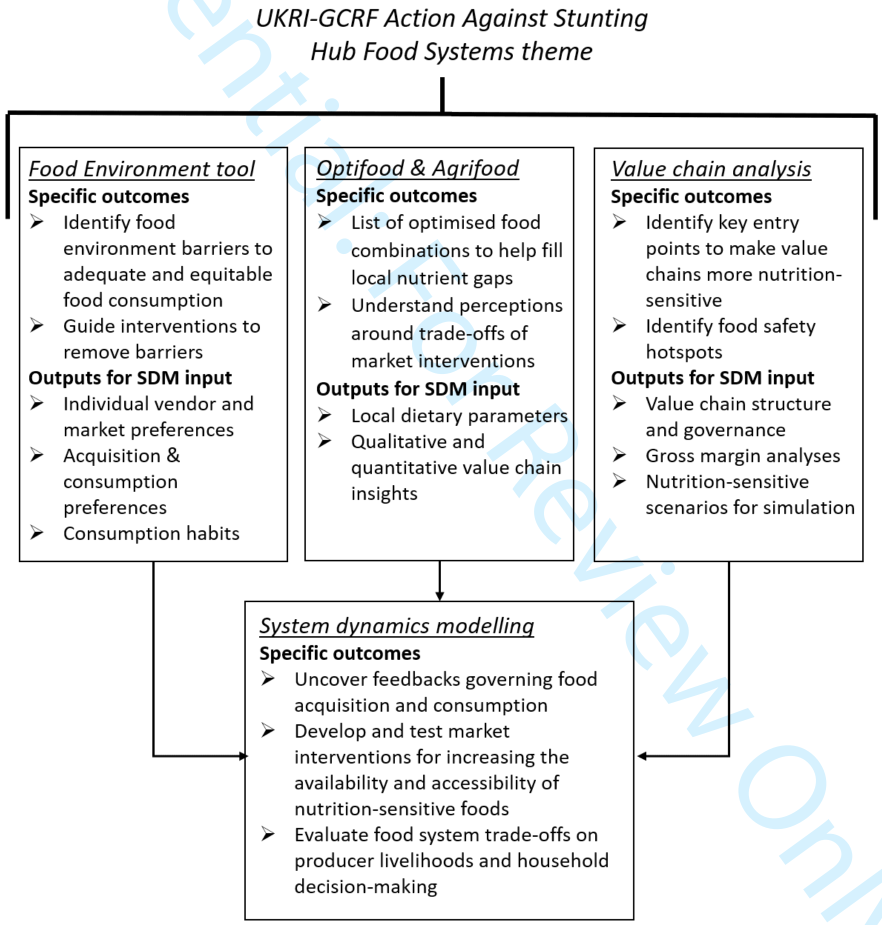

Figure 1: Conceptual framework linking together the sub-studies of the Food Systems theme; SDM = system dynamics modelling.

Characterising food environments

The food environment is the interface that mediates an individual's food acquisition, preparation and consumption.[24] This study will utilise the food environment framework developed by the Agriculture, Nutrition and Health (ANH) Academy Food Environment Working Group [24] to analyse the interactions between the two critical food environment domains (i.e., external supply-side and internal demand-side factors) in the three study

Confidential: For Review Only

communities. Profiling of the supply-side will include mapping distribution networks of nutrient-dense foods (such as green leafy vegetables and animal source foods such as eggs and fish) through key-informant interviews and focus group discussions. However, the limitations are that currently there are no validated metrics to measure food environments in LMICs.[23,24] As a result, we will develop a standardised Food Environment Experience tool to measure the perceived affordability, safety (physical and food safety) and accessibility of food environments across the three study countries.

The tool will be pre-tested in two stages. First, cognitive interviewing [25] will be used to explore the extent to which respondents understand the meaning of key concepts (e.g. *affordability, food acquisition,* and *activity space*). Series of semi-structured interviews with Likert scales and local language translations (i.e., Telugu, French and Bahasa Indonesia) will be conducted. The congnitive questionnaire will first be piloted in a purposively sampled set of respondents not enrolled in the interdisciplinary observational study. In each country, two trained staff will conduct 10 interviews within different demographic (urban and rural) and socio-economic conditions. Transcripts of the interviews will be analysed across two rounds using standard thematic analysis to guide improvements to the wording and framing of questions.[26,27]

Second, the revised questionnaire will be piloted within a purposively sampled set of 30 mothers and children under 2 years of age in each of the study communities. This second testing will again be conducted in mother-infant pairs not included in the observational study. The data generated from the two validation studies will inform the development of a novel Food Environment Experience tool prototype.

The food environment questionnaire will then be administered to mothers enrolled in the observational study at 9 and 12 months postpartum. This questionnaire will uncover insights into why people prioritise particular food environment domains when making food decisions, as well as helping to inform interventions that have the potential to overcome community-level food accessibility barriers.

Optifood and Agrifood modelling to improve food systems

The state-of-the-art *Optifood* and *Agrifood* [28] tools will be used to: (1) identify foods that can address the nutrient gaps in an individual's diet; (2) guide market-based intervention development; (3) identify priority food value chains for assessment; and (4) identify model parameters for the systems dynamic modelling. The model parameters which will be used in the *Optifood* and *Agrifood* tools will be defined using primary data (i.e., dietary intakes) from the observational study, stakeholder workshops, the novel food environment tool and secondary data from Stunting Hub household dietary surveys conducted in the countries.

The analyses will be conducted using linear programming analysis (LPA) in *Optifood* and multi-criteria decision analysis (MCDA) in *Agrifood.* The dietary data obtained from the observational study and dietary surveys (secondary data) will be used in *Optifood* to generate a list of foods and sub-groups of foods consumed in the communities to identify gaps in the nutrient adequacy of maternal and child diets. As a scenario building decision-making tool, *Agrifood* will use these lists, as well as multiple criteria, for decision-making on how to improve the accessibility, availability or affordability of identified foods/food sub-groups via market-based interventions. This will involve conducting workshops with different stakeholders, including community members, value chain stakeholders (e.g., vendors, retailers,

Confidential: For Review Only

traders) and individuals within decision-making organisations (e.g., consumer rights organisations and producer associations). Discussion topics will include how decisions are prioritised in the food system and the value stakeholders place on these criteria. The results of the *Agrifood* analyses will be shared, in another workshop, with these stakeholders to generate discussions related to trade-offs of alternative choices from different stakeholder group perspectives.

Food safety hazards and risks in value chains

Food safety hazards and risks will be assessed along selected value chains for foods with potential to reduce stunting. Commodity selection will be based on the following criteria; a) ability to address nutrition gaps, b) acceptability to consumers, c) acceptability to key value chain stakeholders, and d) amenability to solutions for reduced risk of food-born illnesses.

The observational study will investigate the priority nutrient-dense foods in the study countries. The value chains analysis will be linked to the food environment research to further investigate the sources of nutrient-dense foods (i.e., where people are getting the foods), as well as modes of transportion, storage, processing and consumption. The sources of the food including the point-of-purchase information will be used to link households to a particular food environment. A combination of qualitative and quantitative methodologies, including biological sampling and testing [29,30] will be employed in the value chains for identification of food safety hazards and risks.

It is expected that the priority foods will be delivered by numerous value chains to vulnerable consumers. Thus, the selection of specific value chains to focus on will be based on their contribution to the diets of the target populations, known food safety risks as established in existing literature, and its relevance to stunting in the country context. Based on existing evidence on local diets and food safety risks, likely candidates are fish, milk, and eggs.

Processes, people, products, animals, flows of information and money, practices, regulations, and governance will be assessed for up to five selected value chains per product. This will provide a detailed chain mapping, characterisation of attributes and risks, seasonality, food safety perceptions and behaviours, formal and informal rules, and governing structures.[29,30] Data will be collected through interviews, direct observation, focus group discussions, surveys, and geographical information from key-informants, including primary producers, commission agents and retailers. Food samples of priority value chains at critical chain nodes will be taken and analysed for biological hazards, namely at the following locations: retail and wholesale markets, transport, bulking point (e.g., milk collection centre), harvest, production and pre-harvest inputs. Finally, we will use the information gathered to identify leverage points for cost-effective interventions, to make these foods safer and more available.

Modelling nutrition-sensitive interventions

The resource intensity of standard experimental approaches is a major constraint in estimating the implications of market interventions on child stunting prevention. Furthermore, such experimental designs have limited ability to generate policy-relevant information at scale,[31] including the emergence of trade-offs over time. System dynamics modelling (SDM) is a microsimulation technique which represents system stocks, flows and feedbacks in nonlinear differential equations.[21] Using data collected by the *Agrifood* tool, and food environment and value chain assessments, SDM will be used to conduct 'what-if' scenario analysis to

Confidential: For Review Only

identify potential interventions to improve the availability and affordability of nutrient-dense foods in markets over time.

SDM will also utilise data collected from a campaign of participatory 'group model building' (GMB) in each of the three study sites,[32] which will be focused on discovering the feedbacks underlying household decision-making around food acquisition and consumption. As previously conducted in Bihar and Myanmar,[32] local stakeholders will be first introduced to systems concepts, before participatory discussions on the most important feedbacks. A third session will focus on generating quantitative data to numerically parameterise the models, and then a final session to provide an opportunity to cooperatively evaluate the draft model. The LayerStack offline Geographical Information System (GIS) will be used in the participatory discussions to enable stakeholders to map and visualise system components across space and time.[32]

The sessions will involve up to 15 local stakeholders involved in retail and purchase of nutritient-densef foods, 2-3 facilitators-cum-translators and a specialist modeller to code insights into the modelling software (i.e., STELLA–ISEE systems),[32–34] per country. Discussions will take a focus-group format, whereby the modeller and facilitators guide the discussions through a series of pre-planned topics on the major stocks, flows and feedbacks governing food system dynamics. Purposeful sampling will be used to select participants who are able to participate up to three hours per week, for four weeks. Where socio-culturally appropriate, separate sessions will be held for female and male participants.

The formal model will be quantitatively evaluated against secondary time-series data, and qualitatively with a reference group of 3-4 local experts.[32] Future scenarios will then explore the impacts of nutrition-sensitive interventions (e.g., healthy food promotions or cold storage construction) on the evolution of child stunting-sensitive outcomes, including the seasonal availability of green-leafy vegetables and its distribution and consumption within households.

Data management and analysis

Data will be collected following standardised protocols for value chain mapping, focus group discussions, key-informant interviews and GMB. Interview guides will be developed in English, translated into local languages, and pre-tested before data collection. All the interviews and focus groups will be conducted by trained local research assistants fluent in the local language. Informed consent will be obtained from participants before collecting data and audio-recordings. All participants will be assured of the confidentiality of the questionnaire responses and food sampling data. All primary data will be stored on password protected laptops and desktops in line with central Action Against Stunting Hub protocols.

For the MCDA and LPA, the *Optifood* and *Agrifood* software programmes will be used.[28] Quantitative analysis of the food environment tool, value chain assessments and SDM outputs will be conducted on appropriate statistical software (e.g. STATA and R statistical software); variables will be either continuous, binomial or categorical. Qualitative data from interviews and focus groups will be analysed thematically using coding software (e.g., Nvivo). The quantitative food safety risk assessment will be conducted using existing analytical tools for (1) hazard identification, (2) exposure assessment, (3) dose-response assessment, and (4) risk characterization.

Patient and public involvement

Confidential: For Review Only

Study participants (mothers) and public members (stakeholders and experts) will be involved in value chain interviews, food environment surveys, and GMB. Key local decision-makers will be involved in research dissemination. Partners at the National Institute of Nutrition (NIN-India), Southeast Asian Ministers of Education Organization Regional Center for Food and Nutrition (SEAMEO-RECFON-Indonesia) and University of Cheikh Anta DIOP (Senegal) will facilitate these activities.

ETHICS AND DISSEMINATION

Ethical approval of the study was granted by the institutional ethics committee of the London School of Hygiene and Tropical Medicine (17915/RR/17513), by the Social Science Research Ethical Review Board at the Royal Veterinary College (URN SR2020-0197) and by the International Livestock Research Institute Institutional Research Ethics Committee (ILRI-IREC2020-33). Moreover, in-country ethics approvals were granted by the following bodies: National Institute of Nutrition (ICMR), Ministry of Health and Family Welfare, Government of India (CR/04/I/2021); Health Research Ethics Committee, University of Indonesia and Cipto Mangunkusumo Hospital (KET-887/UN2.F1/ETIK/PPM.00.02/2019); Comité National d'Ethique pour la Recherche en Santé, Senegal (Protocole SEN19/78). Ethical approval for the Food Systems theme was granted by the Ethics Committee of the London School of Hygiene and Tropical Medicine (17919).

Initial findings will be disseminated via seminars, public engagement events, and relevant international conferences. Findings of the study will be published in peer-reviewed journals. Where possible, the publications will encourage authorship of early-career researchers in the research. Finally, the datasets and models generated from the study will be deposited in a public data repository.

Confidential: For Review Only

REFERENCES

1. UNICEF, WHO, World Bank. Levels and trends in child malnutrition: Key findings of the 2020 Edition of the Joint Child Malnutrition Estimates. 2020;:16.https://data.unicef.org/topic/nutrition/malnutrition/

2 Development Initiatives. 2020 Global Nutrition Report: Action on equity to end malnutrition. Bristol, U.K: 2020. https://globalnutritionreport.org/reports/2020-global-nutrition-report/

3 Dewey KG, Begum K. Long-term consequences of stunting in early life. *Matern Child Nutr* 2011;**7 (Suppl 3**:5–18. doi:10.1111/j.1740-8709.2011.00349.x

4 Black RE, Victora CG, Walker SP, *et al.* Maternal and child undernutrition and overweight in low-income and middle-income countries. *Lancet* 2013;**382**:427–51. doi:10.1016/S0140-6736(13)60937-X

5 UN. Transforming our world: the 2030 Agenda for Sustainable Development. 2015. https://sustainabledevelopment.un.org/content/documents/21252030 Agenda for Sustainable Development web.pdf

6 Black RE, Heidkamp R. Causes of Stunting and Preventive Dietary Interventions in Pregnancy and Early Childhood. In: *Nestlé Nutrition Institute Workshop Series*. 2018. 105–13. doi:10.1159/000486496

7 Nshimyiryo A, Hedt-Gauthier B, Mutaganzwa C, *et al.* Risk factors for stunting among children under five years: a cross-sectional population-based study in Rwanda using the 2015 Demographic and Health Survey. *BMC Public Health* 2019;**19**:175. doi:10.1186/s12889-019-6504-z

8 Vaivada T, Akseer N, Akseer S, *et al.* Stunting in childhood: an overview of global burden, trends, determinants, and drivers of decline. *Am J Clin Nutr* 2020;**112**:777S----791S. doi:10.1093/ajcn/nqaa159

9 WHO. WHA Global Nutrition Targets 2025: Stunting Policy Brief. 2012;:10.https://www.who.int/nutrition/topics/globaltargets_stunting_policybrief.pdf

10 de Onis M, Dewey KG, Borghi E, *et al.* The World Health Organization's global target for reducing childhood stunting by 2025: rationale and proposed actions. *Matern Child Nutr* 2013;**9 Suppl 2**:6–26. doi:10.1111/mcn.12075

11 Horton R, Lo S. Nutrition: a quintessential sustainable development goal. *Lancet* 2013;**382**:371–2. doi:10.1016/S0140-6736(13)61100-9

12 Bhutta ZA, Das JK, Rizvi A, *et al.* Evidence-based interventions for improvement of maternal and child nutrition: what can be done and at what cost? *Lancet* 2013;**382**:452–77. doi:10.1016/S0140-6736(13)60996-4

13 Ruel MT, Quisumbing AR, Balagamwala M. Nutrition-sensitive agriculture: What have we learned so far? *Glob Food Sec* 2018;**17**:128–53. doi:https://doi.org/10.1016/j.gfs.2018.01.002

14 Kadiyala S, Prost A, Harris-Fry H, *et al.* Upscaling Participatory Action and Videos for Agriculture and Nutrition (UPAVAN) trial comparing three variants of a nutrition-sensitive agricultural extension intervention to improve maternal and child nutritional outcomes in rural Odisha, India: study p. *Trials* 2018;**19**:176. doi:10.1186/s13063-018-2521-y

15 Pingali P, Sunder N. Transitioning Toward Nutrition-Sensitive Food Systems in Developing Countries. *Annu Rev Resour Econ* 2017;**9**:439–59. doi:10.1146/annurev-resource-100516-053552

16 Ruel MT, Alderman H. Nutrition-sensitive interventions and programmes: how can they help to accelerate progress in improving maternal and child nutrition? *Lancet*

Confidential: For Review Only

2013;**382**:536–51. doi:10.1016/S0140-6736(13)60843-0
17 Heidkamp RA, Piwoz E, Gillespie S, *et al.* Mobilising evidence, data, and resources to achieve global maternal and child undernutrition targets and the Sustainable Development Goals: an agenda for action. *Lancet* 2021;**397**:1400–18. doi:10.1016/S0140-6736(21)00568-7
18 Stifel D, Minten B. Market Access, Well-being, and Nutrition: Evidence from Ethiopia. *World Dev* 2017;**90**:229–41. doi:https://doi.org/10.1016/j.worlddev.2016.09.009
19 Ogutu SO, Qaim M. Commercialization of the small farm sector and multidimensional poverty. *World Dev* 2019;**114**:281–93. doi:https://doi.org/10.1016/j.worlddev.2018.10.012
20 Maestre M, Poole N, Henson S. Assessing food value chain pathways, linkages and impacts for better nutrition of vulnerable groups. *Food Policy* 2017;**68**:31–9. doi:https://doi.org/10.1016/j.foodpol.2016.12.007
21 Cooper GS, Shankar B, Rich KM, *et al.* Can fruit and vegetable aggregation systems better balance improved producer livelihoods with more equitable distribution? *World Dev* 2021;**148**:105678. doi:https://doi.org/10.1016/j.worlddev.2021.105678
22 Rammelt CF, Leung MWH. Tracing the Causal Loops Through Local Perceptions of Rural Road Impacts in Ethiopia. *World Dev* 2017;**95**:1–14. doi:https://doi.org/10.1016/j.worlddev.2017.02.024
23 Turner C, Kalamatianou S, Drewnowski A, *et al.* Food Environment Research in Low- and Middle-Income Countries: A Systematic Scoping Review. *Adv Nutr* 2020;**11**:387–97. doi:10.1093/advances/nmz031
24 Turner C, Aggarwal A, Walls H, *et al.* Concepts and critical perspectives for food environment research: A global framework with implications for action in low- and middle-income countries. *Glob Food Sec* 2018;**18**:93–101. doi:https://doi.org/10.1016/j.gfs.2018.08.003
25 Beatty PC, Willis GB. Research Synthesis: The Practice of Cognitive Interviewing. *Public Opin Q* 2007;**71**:287–311. doi:10.1093/poq/nfm006
26 Ashok S, Kim SS, Heidkamp RA, *et al.* Using cognitive interviewing to bridge the intent-interpretation gap for nutrition coverage survey questions in India. *Matern Child Nutr* 2021;**n/a**:e13248. doi:https://doi.org/10.1111/mcn.13248
27 Scott K, Gharai D, Sharma M, *et al.* Yes, no, maybe so: the importance of cognitive interviewing to enhance structured surveys on respectful maternity care in northern India. *Health Policy Plan* 2020;**35**:67–77. doi:10.1093/heapol/czz141
28 Knight F, Ferguson E. Agrifood; a new Multi-Criteria Decision Analysis tool to examine trade-offs in programme decisions for nutrition sensitive agriculture. In: *Agriculture, Nutrition and Health Academy Week*. Hyderabad, India: 2019. 18.https://www.anh-academy.org/sites/default/files/7A_Frances
29 Aslam H Bin, Alarcon P, Yaqub T, *et al.* A Value Chain Approach to Characterize the Chicken Sub-sector in Pakistan. Front. Vet. Sci. 2020;**7**:361.https://www.frontiersin.org/article/10.3389/fvets.2020.00361
30 Alarcon P, Fèvre EM, Murungi MK, *et al.* Mapping of beef, sheep and goat food systems in Nairobi — A framework for policy making and the identification of structural vulnerabilities and deficiencies. *Agric Syst* 2017;**152**:1–17. doi:https://doi.org/10.1016/j.agsy.2016.12.005
31 Deaton A, Cartwright N. Understanding and misunderstanding randomized controlled trials. *Soc Sci Med* 2018;**210**:2–21. doi:https://doi.org/10.1016/j.socscimed.2017.12.005
32 Rich KM, Berends J, Cooper GS. Enriching value chains through maps: reflections

Confidential: For Review Only

from spatial group model building in Myanmar and India. *Dev Pract* 2021;:1–7. doi:10.1080/09614524.2021.1907545

33 Tako AA, Robinson S. Model development in discrete-event simulation and system dynamics: An empirical study of expert modellers. *Eur J Oper Res* 2010;**207**:784–94. doi:10.1016/j.ejor.2010.05.011

34 Cooper GS, Rich KM, Shankar B, *et al.* Identifying 'win-win-win'' futures from inequitable value chain trade-offs: A system dynamics approach'. *Agric Syst* 2021;**190**. doi:10.1016/j.agsy.2021.103096

Confidential: For Review Only

Authors contributions:

Gregory S Cooper: Design – development of methods; Writing – led original draft, editing, final version

Hilary Davies-Kershaw: Design – development of methods; Writing – editing, final version

Paula Dominguez-Salas: Design – study conceptualisation, development of methods; Writing – contribution to original draft, editing, final version

Umi Fahmida: Design – study conceptualisation, development of methods; Writing – contribution to original draft, editing

Babacar Faye: Design – study conceptualisation; Writing – editing

Elaine L Ferguson: Design – study conceptualisation, development of methods; Writing – contribution to original draft, editing, final version

Delia Grace: Design – development of methods; Writing – editing

Barbara N Häsler: Design – study conceptualisation, development of methods; Writing – contribution to original draft, editing, final version

Suneetha Kadiyala: Design – study conceptualisation, development of methods; Writing – contribution to original draft, editing, final version

Archana Konapur: Design – development of methods; Writing – editing

Bharati Kulkarni: Design – study conceptualisation, development of methods; Writing – contribution to original draft, editing

Bhagyalakshmi C Prakashbabu: Design – development of methods; Writing – editing

Indriya L Pramesthi: Design – development of methods; Writing – editing

Dominic Rowland: Design – study conceptualisation, development of methods; Writing – contribution to original draft, editing

Kirtuhika Selvaraj: Design – development of methods; Writing – contribution to original draft, editing

Bhavani Shankar: Design – study conceptualisation, development of methods; Writing – contribution to original draft, editing, final version

Arienta R P Sudibya: Design – development of methods; Writing – editing

Roger C Tine: Design – study conceptualisation, development of methods; Writing – editing

Dinesh Yadav: Design – study conceptualisation, development of methods; Writing – editing

Nur L Zahra: Design – development of methods; Writing – contribution to original draft, editing

Claire Heffernan: Design - study conceptualisation; Writing - editing

Funding: This study was funded by UK Research and Innovation Global Challenges Research Fund (UKRI-GCRF) (Project ref. MR/S01313X/1). The views expressed in this work are those of the authors and not the funder.

Conflict of interest statement: The authors declare no conflict of interest.

Confidential: For Review Only

Acknolwedgements: Thank you to Dr Modou Jobarteh and Kaitlin Conway for their kind and constructive comments during the preparation of this manuscript.

Confidential: For Review Only

BMJ Paediatrics Open

Investigating market-based opportunities for the provision of nutritious and safe diets to prevent childhood stunting: a UKRI-GCRF Action Against Stunting Hub protocol paper

Journal:	*BMJ Paediatrics Open*
Manuscript ID	bmjpo-2022-001671.R1
Article Type:	Protocol
Date Submitted by the Author:	11-Nov-2022
Complete List of Authors:	Cooper, Gregory; The University of Sheffield, Institute of Sustainable Food and Department of Geography<br>Davies-Kershaw, Hilary; London School of Hygiene and Tropical Medicine Faculty of Epidemiology and Public Health, Department of Population Health<br>Dominguez-Salas, Paula; International Livestock Research Institute; University of Greenwich, Natural Resources Institute<br>Fahmida, Umi; Southeast Asian Ministers of Education Organization Regional Centre for Food and Nutrition<br>Faye, Babacar; Cheikh Anta Diop University of Dakar, Department of Parasitology-Mycology<br>Ferguson, Elaine; London School of Hygiene and Tropical Medicine Faculty of Epidemiology and Public Health<br>Grace, Delia; International Livestock Research Institute<br>Häsler, Barbara N; The Royal Veterinary College Department of Pathobiology and Population Sciences<br>Kadiyala, Suneetha; London School of Hygiene and Tropical Medicine Faculty of Epidemiology and Population Health, Department of Population Health<br>Konapur, Archana; ICMR National Institute of Nutrition<br>Kulkarni, Bharati; ICMR National Institute of Nutrition<br>Chengat Prakashbabu, Bhagyalakshmi ; The Royal Veterinary College Department of Pathobiology and Population Sciences<br>Pramesthi, Indriya ; Southeast Asian Ministers of Education Organization Regional Centre for Food and Nutrition<br>Rowland, Dominic; Center for International Forestry Research; SOAS, Centre for Environment, Development and Policy (CeDEP)<br>Selvaraj, Kiruthika; ICMR National Institute of Nutrition<br>Sudibya, Arienta R.P.; Southeast Asian Ministers of Education Organization Regional Centre for Food and Nutrition<br>Tine, Roger ; Cheikh Anta Diop University of Dakar, Department of Parasitology-Mycology<br>Yadav, Dinesh; ICMR National Institute of Nutrition<br>Zahra, Nur ; Southeast Asian Ministers of Education Organization Regional Centre for Food and Nutrition<br>Shankar, Bhavani; The University of Sheffield, Institute of Sustainable Food and Department of Geography<br>Heffernan, Claire; The Royal Veterinary College Department of

	Pathobiology and Population Sciences; London International Development Centre
Keywords:	Nutrition

SCHOLARONE™
Manuscripts

I, the Submitting Author has the right to grant and does grant on behalf of all authors of the Work (as defined in the below author licence), an exclusive licence and/or a non-exclusive licence for contributions from authors who are: i) UK Crown employees; ii) where BMJ has agreed a CC-BY licence shall apply, and/or iii) in accordance with the terms applicable for US Federal Government officers or employees acting as part of their official duties; on a worldwide, perpetual, irrevocable, royalty-free basis to BMJ Publishing Group Ltd ("BMJ") its licensees and where the relevant Journal is co-owned by BMJ to the co-owners of the Journal, to publish the Work in this journal and any other BMJ products and to exploit all rights, as set out in our licence.

The Submitting Author accepts and understands that any supply made under these terms is made by BMJ to the Submitting Author unless you are acting as an employee on behalf of your employer or a postgraduate student of an affiliated institution which is paying any applicable article publishing charge ("APC") for Open Access articles. Where the Submitting Author wishes to make the Work available on an Open Access basis (and intends to pay the relevant APC), the terms of reuse of such Open Access shall be governed by a Creative Commons licence – details of these licences and which Creative Commons licence will apply to this Work are set out in our licence referred to above.

Other than as permitted in any relevant BMJ Author's Self Archiving Policies, I confirm this Work has not been accepted for publication elsewhere, is not being considered for publication elsewhere and does not duplicate material already published. I confirm all authors consent to publication of this Work and authorise the granting of this licence.

Confidential: For Review Only

Investigating market-based opportunities for the provision of nutritious and safe diets to prevent childhood stunting: a UKRI-GCRF Action Against Stunting Hub protocol paper

Gregory S Cooper[1], Hilary Davies-Kershaw[2], Paula Dominguez-Salas[3,4], Umi Fahmida[5], Babacar Faye[6], Elaine L Ferguson[2], Delia Grace Randolph[3], Barbara N Häsler[7], Suneetha Kadiyala[2], Archana Konapur[8], Bharati Kulkarni[8], Bhagyalakshmi C Prakashbabu[7], Indriya L Pramesthi[5], Dominic Rowland[9, 10], Kiruthika Selvaraj[8], Arienta R P Sudibya[5], Roger C Tine[6], Dinesh Yadav[8], Nur L Zahra[5], Bhavani Shankar[1] and Claire Heffernan[11]

Affiliations

[1]Institute for Sustainable Food and Department of Geography, University of Sheffield, Sheffield, United Kingdom

[2] Department of Population Health, London School of Hygiene and Tropical Medicine, London, United Kingdom

[3]International Livestock Research Institute (ILRI), Nairobi, Kenya

[4]Natural Resources Institute, University of Greenwich, London, United Kingdom

[5] Southeast Asian Ministers of Education Organization Regional Center for Food and Nutrition (SEAMEO-RECFON)-Pusat Kajian Gizi Regional, Universitas Indonesia, Jakarta, Indonesia

[6] Department of Parasitology-Mycology, University of Cheikh Anta DIOP, Dakar, Senegal

[7]Department of Pathobiology and Population Sciences, Royal Veterinary College (RVC), Hatfield, United Kingdom

[8]ICMR- National Institute of Nutrition (ICMR), Hyderabad, Telangana, India

[9] Center for International Forestry Research, Bogor, Indonesia

Confidential: For P

[10] Centre for Development, Environment and Policy (CeDEP), SOAS University of London, London, United Kingdom

[11] London International Development Centre (LIDC), London, United Kingdom

Corresponding author:

Dr Gregory S Cooper

Email: g.s.cooper@sheffield.ac.uk

Confidential: For Review Only

ABSTRACT

Background

Inadequate access to affordable, safe, desirable and convenient nutrient-dense food is one of the underlying causes of child stunting. Whilst targeted nutrition-sensitive interventions (e.g., backyard ‘nutri-gardens’) may increase dietary diversity within farming households, such interventions have limited scalability across the wider food system where markets remain underdeveloped. This research aims to develop and assess market-based interventions for key nutrient-dense foods to help improve the diets of women and children in the first 1000 days of life.

Methods

Data collection uses four parallel approaches in each of the three study countries (India, Indonesia and Senegal). (1) A novel *food environment tool* will be developed to characterise the accessibility and affordability of nutrient-dense foods in the study countries. The tool will be validated through pre-testing using cognitive interviewing and piloting in purposively sampled households, 10 (cognitive interviewing) and 30 (piloting) households in each country. (2) Stakeholder interviews (e.g., with producers, intermediaries and retailers) will be conducted to map out nutrition-sensitive entry points of key value chains (e.g., animal-sourced foods), before hotspots of potential food safety hazards will be identified from food samples collected along the chains. (3) The *Optifood* and *Agrifood* tools will be used to identify foods that can address food system nutrient gaps and engage key stakeholders to prioritise market interventions to improve nutrition outcomes. *Optifood* and *Agrifood* parameters will be informed by publicly available data, plus interviews and focus groups with value chain stakeholders. (4) Informed by the previous three approaches and a campaign of

Confidential: For Review Only

53 participatory 'group model building', a novel system dynamics model will evaluate the
54 impact of alternative market-based solutions on the availability and affordability of nutrient-
55 dense foods over time.
56
57 **Ethics and dissemination**
58 The study has received ethical approval in the United Kingdom, Senegal, Indonesia and
59 India. Dissemination comprises peer-reviewed journals, international disciplinary
60 conferences, and multi-stakeholder dissemination workshops.
61
62

Confidential: For Review Only

What is already known on this topic

- Access to nutrient-dense food that is affordable, acceptable, safe, and convenient is a prerequisite to counteracting food and nutrition insecurity in early life.
- In countries such as Indonesia, India and Senegal, where the ability to meet this decade's international stunting targets remains uncertain, increasing attention is being paid to the nutrition-sensitivity of market-based interventions that help to improve food storage, safety and equitable distribution.

What this study hopes to add

- Develop and apply a novel food environment profiling tool, value chains food safety analysis, and the *Optifood* and *Agrifood* models, to capture the complex socioeconomic and institutional barriers to equitable nutrition security.
- Utilise systems-based modelling to evaluate the wider socioeconomic trade-offs and unintended consequences of nutrition-sensitive market interventions.

How this study might affect research, practice or policy

- This study will assess and recommend tailored market-based interventions within the three countries to guide decision-making around improving women and children's year-round access to locally relevant, nutrient-dense food.
-

Confidential: For Review Only

INTRODUCTION

As of early-2020, stunting affected approximately 149-million children under the age of five years, with 65% of all stunted children living in low- and middle-income countries (LMICs).[1,2] The inability to achieve optimal linear growth in the early years of life is associated with a spectrum of development barriers, including the reduced ability to reach full cognitive potential, inferior rates of school achievement, economic productivity and maternal reproductive outcomes.[1,3,4] Child stunting reaches across eight of the 17 Sustainable Development Goals (SDGs).[5] The causes of child stunting are widely understood to be multidimensional, associated with the interactions between poverty, hunger, gender inequity, poor health, poor sanitation and poor quality education, as well as an outcome related to physically taxing work and wider structural inequalities.[6–8]

Reflecting the complex and pernicious nature of the problem, the World Health Assembly (WHA) in 2012 recognized child stunting as 'one of the most significant impediments to human development' and resolved to decrease the number of children under five who are stunted by 40% by 2025.[9] However, even prior to the COVID-19 pandemic, progress around stunting reduction was acknowledged to be slowing,[2,10] with researchers noting in 2013 that the WHA target was already on course to be missed.[11]

In an effort to evaluate the contributions of targeted interventions towards stunting reduction, the 2013 Lancet Series on Maternal and Child Nutrition found that even achieving high coverage (90%) across 10 recommended nutrition-specific interventions will likely only reduce the stunting burden in LMICs by 20%.[4,12] Numerous studies have since promoted nutrition-sensitive strategies to provide the foundations of food and livelihood security, improved diet quality and women's empowerment,[13–15] upon which synergistic nutrition-specific

Confidential: For Review Only

interventions can be scaled-up to help tackle stunting.[13,16,17] In particular, increasing attention is being paid to the nutritional sensitivity of markets,[18–20] and there is growing understanding that improved rural connectivity, cold storage and processing facilities, and accessible and safe food environments may improve the equitable distribution, pricing and consumption of nutritious foods.[21–23] Enhancing the quality of diets by harnessing such market-based solutions may be beneficial to the reduction of the risk of child stunting.

Therefore, as part of the UKRI-GCRF Action Against Stunting Hub (Jobarteh et al., *this issue*), a project which aims to understand the contributions of multidimensional drivers to child stunting (e.g., gut health, nutrition, sanitation, education and the home environment), this particular study to focus on the role of the food system to develop and assess the potential of tailored market interventions with key nutrient-dense foods (e.g., animal source foods, fruits and vegetables) to improve the diet and nutrition of pregnant and lactating women and children. Therefore, the specific objectives of the food systems component of the Action Against Stunting study are:

1. To characterise the food environments in India, Indonesia and Senegal to assess the *what*, *when*, *where* and *how* of food acquisition and consumption, with a focus on children under two years of age.
2. To refine and apply tools based on linear programming analyses (LPA) and multiple criteria decision analysis (MCDA) to guide decisions on how to make markets more nutrition-sensitive.
3. To identify critical food safety risks and hazards in food value chains with the potential for child stunting alleviation.
4. To design nutrition-sensitive market interventions and investigate their potential to deliver nutritious foods under various future scenarios.

Confidential: For Review Only

METHODS AND ANALYSIS

Study design

This study is part of an observational research programme into understanding the myriad of factors contributing to poor growth and development in the first 1000 days of childhood. The interdisciplinary research recruits women during pregnancy in India (Hyderabad), Indonesia (Lombok) and Senegal (Kaffrine) and follows mother-infant pairs up to 24 months after birth, with detailed investigations at predefined timepoints to support analysis into the aetiology of child stunting. This study will provide an understanding of the food environments in the study countries and its likely contribution to child stunting. Specific tools will be developed to provide in-depth analyses of the food systems including household food environments, local markets, and priority value chains, with a view to informing programmes and policies on strategies to meet the nutrient gaps in maternal and child diets. The conceptual framework - consisting of three parallel data collections which all feed into the construction of a systems dynamic model - is depicted in Figure 1.

Characterising food environments

The food environment is the interface that mediates an individual's food acquisition, preparation and consumption.[24] This study will utilise the food environment framework developed by the Agriculture, Nutrition and Health (ANH) Academy Food Environment Working Group [24] to analyse the interactions between the two critical food environment

[PLEASE INSERT FIGURE 1 HERE]

Figure 1: Conceptual framework linking together the sub-studies of the Food Systems theme; SDM = system dynamics modelling. As denoted by the arrows, the quantitative and qualitative information generated by the three parallel data collections (Food Environment tool, Optifood and Agrifood models, and value chain analysis) will directly inform the design of the system dynamics modelling activities.

Confidential: For Review Only

domains (i.e., external supply-side and internal demand-side factors) in the three study communities.

As detailed in the value chain mapping activity below, profiling of the supply-side will include mapping distribution networks of key target nutrient-dense foods (such as dark green leafy vegetables and animal source foods such as eggs and fish) through key-informant interviews and focus group discussions. However, at present, there are no validated metrics to measure the perception of people towards their local food environments in LMICs.[23,24] As a result, we will develop a standardised Food Environment Experience tool to measure the perceived affordability, safety (physical and food safety), and physical and financial accessibility of food environments across the three study locations (see Table 1 for a full list of food environment concepts captured by the tool).

The tool will be pre-tested in two stages. First, cognitive interviewing [25] will be used to explore the extent to which respondents understand the meaning of key concepts (e.g. *affordability, food acquisition,* and *activity space*). Series of semi-structured interviews with Likert scales and local language translations (i.e., Telugu, French and Bahasa Indonesia) will be conducted. The cognitive questionnaire will first be piloted in a purposively sampled set of respondents not enrolled in the interdisciplinary observational study. In each country, two trained staff will conduct 10 interviews within different demographic (urban and rural) and socio-economic conditions. Transcripts of the interviews will be analysed across two rounds using standard thematic analysis to guide improvements to the wording and framing of questions.[26,27]

Confidential: For Review Only

Second, the revised questionnaire will be piloted within a purposively sampled set of 30 mothers and children under 2 years of age in each of the study communities. This second testing will again be conducted in mother-infant pairs not included in the observational study. The data generated from the two validation studies will inform the development of a novel Food Environment Experience tool prototype.

The food environment questionnaire will then be administered to mothers enrolled in the observational study at 9 and 12 months postpartum. With the recruitment of cohort households happening at over the course of one year, staggering the data collection across successive months will provide insights into how the availability and affordability of nutrient-dense foods

Table 1: Descriptions of the food environment concepts captured in the Food Environment Experience tool, built on the food environment dimensions and domains detailed in Turner et al. (2018). Dark green leafy vegetables = DGLVs.

**Dimension**	**Domain**	**Concept in the Food Environment Experience tool**	**Sample question from Food Environment Experience tool**
Activity space	Personal & external	The places that household members visit as a part of their daily routine	*In the last 30 days, what are all the places from which you or other household members have visited, as a part of your daily routine, to get food for household members to consume?*
Availability	External	Food product was observed to be present by the respondent	*In the last 30 days, in the places you visit as a part of your daily routine, how often were DGLVs available?*
Vendor properties	External	Potential to acquire food product on credit	*If you did try to get DGLVs on credit with written agreement during the last 30 days, how often was it possible to do so successfully?*
	External	Food quality, comprised of food safety and hygiene	*Thinking of all the places where DGLVs were available in the last 30 days, how often did you see DGLVs that you would consider hygienic (regardless of whether you got them or not)?*
Marketing and regulation	External	Promotional information (e.g., nutritional messages, adverts, special offers)	*During the last 30 days, how often did you read, hear, or watch advertisements to buy DGLVs?*

Confidential: For Review Only

Physical accessibility	Personal & external	Experience of physical safety and security	*In the last 30 days, how often did you find dark DGLVs in areas that you would consider safe to go to?*
	Personal & external	Ease of physical access	*In the last 30 days, how often did you find DGLVs in places that are easy to go to?*
Affordability	Personal & external	Food product priced at a cost that the household could spend	*During the last 30 days, in the places you have visited as a part of your daily routine, how often did you find DGLVs that were priced at a cost that your household could spend?*
Convenience	Personal	Sufficient time to acquire, prepare and cook food product	*In your opinion, were there any times in the last 30 days when you or your household members did not have sufficient time to get DGLVs for household consumption? If yes, how often did you NOT have sufficient time to get DGLVs?*
Desirability	Personal	Tastes, taboos and cultural compatibility, health attitudes and knowledge	*Do people who are important to you (e.g. family/friend) like the taste of DGLVs? If yes, how strongly do they feel?*

varies seasonally (i.e., 12 successive months of data from May 2022 to April 2023 for dark green leafy vegetables in Hyderabad). In turn, this questionnaire will uncover insights into why people prioritise particular food environment domains when making food decisions, as well as helping to inform interventions that have the potential to overcome community-level food accessibility barriers.

Optifood and Agrifood modelling to improve food systems

The state-of-the-art Optifood and Agrifood [28] tools will be used to: (1) identify foods that can address the nutrient gaps in an individual's diet; (2) guide market-based intervention development; (3) identify priority food value chains for assessment; and (4) identify model parameters for the systems dynamic modelling. The model parameters which will be used in the Optifood and Agrifood tools will be defined using primary data (i.e., dietary intakes) from the observational study, stakeholder workshops, the novel food environment tool and secondary data from Stunting Hub household dietary surveys conducted in the countries. All

Confidential: For Review Only

dietary intakes in the AASH observation study will be collected using the four-pass 24-hour (MP24HR) dietary recall method [29]. Maternal intakes will be collected during the second and third trimesters in East Lombok, Indonesia and Kaffrine, Senegal and during the third trimester in Hyderabad, India. Infant dietary intake will be collected at 6, 9, 12, 18 and 24 months after birth. A second MP24HR will be collected from a 10% random sub-sample from each country site at each time-point to reduce random measurement error [30].

The analyses will be conducted using linear programming analysis (LPA) in *Optifood* and multi-criteria decision analysis (MCDA) in *Agrifood*. The dietary data obtained from the observational study and dietary surveys (secondary data) will be used in *Optifood* to generate a list of foods and sub-groups of foods consumed in the communities to identify gaps in the nutrient adequacy of maternal and child diets. As a scenario building decision-making tool, *Agrifood* will use these lists, as well as multiple criteria, for decision-making on how to improve the accessibility, availability or affordability of identified foods/food sub-groups via market-based interventions. This will involve conducting workshops with different stakeholders, including community members, value chain stakeholders (e.g., vendors, retailers, traders) and individuals within decision-making organisations (e.g., consumer rights organisations and producer associations). Discussion topics will include how decisions are prioritised in the food system and the value stakeholders place on these criteria. The results of the *Agrifood* analyses will be shared, in another workshop, with these stakeholders to generate discussions related to trade-offs of alternative choices from different stakeholder group perspectives.

Food safety hazards and risks in value chains

Confidential: For Review Only

Food safety hazards and risks will be assessed along selected value chains for foods with potential to reduce stunting. Commodity selection will be based on the following criteria; a) ability to address nutrition gaps, b) acceptability to consumers, c) acceptability to key value chain stakeholders, and d) amenability to solutions for reduced risk of food-born illnesses.

The observational study will investigate the priority nutrient-dense foods in the study countries. The value chains analysis will be linked to the food environment research to further investigate the sources of nutrient-dense foods (i.e., where people are getting the foods), as well as modes of transportation, storage, processing and consumption. The sources of the food including the point-of-purchase information will be used to link households to a particular food environment. A combination of qualitative and quantitative methodologies, including biological sampling and testing [31,32] (specifically for pathogens such as E.coli, Salmonella, Shigella, and Campylobacter), will be employed in the value chains for identification of food safety hazards and risks.

It is expected that the priority foods will be delivered by numerous value chains to vulnerable consumers. Thus, the selection of specific value chains to focus on will be based on their contribution to the diets of the target populations, known food safety risks as established in existing literature, and its relevance to stunting in the country context. Based on existing evidence on local diets and food safety risks, likely candidates are fish, milk, and eggs.

Processes, people, products, animals, flows of information and money, practices, regulations, and governance will be assessed for up to five selected value chains per product. This will provide a detailed chain mapping, characterisation of attributes and risks, seasonality, food safety perceptions and behaviours, formal and informal rules, and governing structures.[31,32]

Confidential: For Review Only

Data will be collected through interviews, direct observation, focus group discussions, surveys, and geographical information from key-informants, including primary producers, commission agents and retailers. High-level representatives will be also interviewed, such as those from relevant Ministries or the Food Safety authority. Food samples of priority value chains at critical chain nodes will be taken and analysed for biological hazards, namely at the following locations: retail and wholesale markets, transportation, bulking point (e.g., milk collection centre), harvest, production and pre-harvest inputs. Finally, we will use the information gathered to identify leverage points for cost-effective interventions, to make these foods safer and more available.

Modelling nutrition-sensitive interventions

The resource intensity of standard experimental approaches is a major constraint in estimating the implications of market interventions on child stunting prevention. Furthermore, such experimental designs have limited ability to generate policy-relevant information at scale,[33] including the emergence of trade-offs over time. System dynamics modelling (SDM) is a microsimulation technique which represents system stocks, flows and feedbacks in nonlinear differential equations.[21] Using data collected by the *Agrifood* tool, and food environment and value chain assessments, SDM will be used to conduct 'what-if' scenario analysis to identify potential interventions to improve the availability and affordability of nutrient-dense foods in markets over time.

SDM will also utilise data collected from a campaign of participatory 'group model building' (GMB) in each of the three study sites,[34] which will be focused on discovering the feedbacks underlying household decision-making around food acquisition and consumption. As previously conducted in Bihar and Myanmar,[34] local stakeholders will be first introduced to

Confidential: For Review Only

systems concepts, before participatory discussions on the most important feedbacks. A third session will focus on generating quantitative data to numerically parameterise the models, and then a final session to provide an opportunity to cooperatively evaluate the draft model. The LayerStack offline Geographical Information System (GIS) will be used in the participatory discussions to enable stakeholders to map and visualise system components across space and time.[34]

The sessions will involve up to 15 local stakeholders involved in retail and purchase of nutrient-dense foods, 2-3 facilitators-cum-translators and a specialist modeller to code insights into the modelling software (i.e., STELLA–ISEE systems),[34–36] per country. Discussions will take a focus-group format, whereby the modeller and facilitators guide the discussions through a series of pre-planned topics on the major stocks, flows and feedbacks governing food system dynamics. Purposeful sampling will be used to select participants who are able to participate up to three hours per week, for four weeks. Where socio-culturally appropriate, separate sessions will be held for female and male participants.

The formal model will be quantitatively evaluated against secondary time-series data, and qualitatively with a reference group of 3-4 local experts.[34] Future scenarios will then explore the impacts of nutrition-sensitive interventions (e.g., healthy food promotions or cold storage construction) on the evolution of child stunting-sensitive outcomes, including the seasonal availability of our target food item of interest (e.g., dark green leafy vegetables), and its distribution and consumption within households.

Data management and analysis

Confidential: For Review Only

Data will be collected following standardised protocols for value chain mapping, focus group discussions, key-informant interviews and GMB. Interview guides will be developed in English, translated into local languages, and pre-tested before data collection. All the interviews and focus groups will be conducted by trained local research assistants fluent in the local language. Informed consent will be obtained from participants before collecting data and audio-recordings. All participants will be assured of the confidentiality of the questionnaire responses and food sampling data. All primary data will be stored on password protected laptops and desktops in line with central Action Against Stunting Hub protocols.

For the MCDA and LPA, the *Optifood* and *Agrifood* software programmes will be used.[28] Qualitative data from value chain interviews and focus groups will be analysed thematically using coding software (e.g., Nvivo). Quantitative analysis of the food environment tool, value chain assessments and SDM outputs will be conducted using appropriate statistical software (e.g. STELLA-ISEE Systems and R statistical software). Analysis of the Food Environment Experience tool will focus on three dimensions. First, to both compare between food products and locations at an aggregated level, summary statistics such as weighted averages (i.e., weighted by the number of households which visited each source to obtain food for household consumption) and measures of variance will describe the overall levels of food availability, affordability and experiences of individual food environment dimensions (Table 1). Second, standard correlation analysis (e.g., Peason's r) and linear regression modelling will explore whether potential links exist between the food environment experiences of individual households and their food acquisition behaviours over the last 30 days. Third, linear dimensionality reduction techniques, such as Principal Component Analysis and Factor Analysis, will explore the importance of individual subdomains towards the overall food

Confidential: For Review Only

environment experience dimensions (e.g., convenience of food procurement versus convenience of food preparation).

In turn, both the validation and simulation of the SDM model will utilise standard Monte Carlo techniques to (a) understand the extent to which the model can replicate historical patterns of system behaviour (e.g., seasonal dynamics in NDF availability),[37] and (b) explore the extent to which uncertainties in model design and parameterisation influence the reliability of model projections.[36]

Patient and public involvement

Study participants (mothers) and public members (stakeholders and experts) will be involved in value chain interviews, food environment surveys, and GMB. Key local decision-makers will be involved in research dissemination. Partners at the National Institute of Nutrition (NIN-India), Southeast Asian Ministers of Education Organization Regional Center for Food and Nutrition (SEAMEO-RECFON-Indonesia) and University of Cheikh Anta DIOP (Senegal) will facilitate these activities.

ETHICS AND DISSEMINATION

Ethical approval of the study was granted by the institutional ethics committee of the London School of Hygiene and Tropical Medicine (17915/RR/17513), by the Social Science Research Ethical Review Board at the Royal Veterinary College (URN SR2020-0197) and by the International Livestock Research Institute Institutional Research Ethics Committee (ILRI-IREC2020-33). Moreover, in-country ethics approvals were granted by the following bodies: National Institute of Nutrition (ICMR), Ministry of Health and Family Welfare, Government

https://mc.manuscriptcentral.com/bmjpo

Confidential: For Review Only

of India (CR/04/I/2021); Health Research Ethics Committee, University of Indonesia and Cipto Mangunkusumo Hospital (KET-887/UN2.F1/ETIK/PPM.00.02/2019); Comité National d'Ethique pour la Recherche en Santé, Senegal (Protocole SEN19/78). Ethical approval for the Food Systems theme was granted by the Ethics Committee of the London School of Hygiene and Tropical Medicine (17919).

Initial findings will be disseminated via seminars, public engagement events, and relevant international conferences. Findings of the study will be published in peer-reviewed journals. Where possible, the publications will encourage authorship of early-career researchers in the research. Finally, the datasets and models generated from the study will be deposited in a public data repository.

Confidential: For Review Only

REFERENCES

1 UNICEF, WHO, World Bank. Levels and trends in child malnutrition: Key findings of the 2020 Edition of the Joint Child Malnutrition Estimates. 2020;:16.https://data.unicef.org/topic/nutrition/malnutrition/

2 Development Initiatives. 2020 Global Nutrition Report: Action on equity to end malnutrition. Bristol, U.K: 2020. https://globalnutritionreport.org/reports/2020-global-nutrition-report/

3 Dewey KG, Begum K. Long-term consequences of stunting in early life. *Matern Child Nutr* 2011;**7 (Suppl 3**:5–18. doi:10.1111/j.1740-8709.2011.00349.x

4 Black RE, Victora CG, Walker SP, *et al.* Maternal and child undernutrition and overweight in low-income and middle-income countries. *Lancet* 2013;**382**:427–51. doi:10.1016/S0140-6736(13)60937-X

5 UN. Transforming our world: the 2030 Agenda for Sustainable Development. 2015. https://sustainabledevelopment.un.org/content/documents/21252030

6 Black RE, Heidkamp R. Causes of Stunting and Preventive Dietary Interventions in Pregnancy and Early Childhood. In: *Nestlé Nutrition Institute Workshop Series*. 2018. 105–13. doi:10.1159/000486496

7 Nshimiyiryo A, Hedt-Gauthier B, Mutaganzwa C, *et al.* Risk factors for stunting among children under five years: a cross-sectional population-based study in Rwanda using the 2015 Demographic and Health Survey. *BMC Public Health* 2019;**19**:175. doi:10.1186/s12889-019-6504-z

8 Vaivada T, Akseer N, Akseer S, *et al.* Stunting in childhood: an overview of global burden, trends, determinants, and drivers of decline. *Am J Clin Nutr* 2020;**112**:777S----791S. doi:10.1093/ajcn/nqaa159

9 WHO. WHA Global Nutrition Targets 2025: Stunting Policy Brief. 2012;:10.https://www.who.int/nutrition/topics/globaltargets_stunting_policybrief.pdf

10 de Onis M, Dewey KG, Borghi E, *et al.* The World Health Organization's global target for reducing childhood stunting by 2025: rationale and proposed actions. *Matern Child Nutr* 2013;**9 Suppl 2**:6–26. doi:10.1111/mcn.12075

11 Horton R, Lo S. Nutrition: a quintessential sustainable development goal. *Lancet* 2013;**382**:371–2. doi:10.1016/S0140-6736(13)61100-9

12 Bhutta ZA, Das JK, Rizvi A, *et al.* Evidence-based interventions for improvement of maternal and child nutrition: what can be done and at what cost? *Lancet* 2013;**382**:452–77. doi:10.1016/S0140-6736(13)60996-4

13 Ruel MT, Quisumbing AR, Balagamwala M. Nutrition-sensitive agriculture: What have we learned so far? *Glob Food Sec* 2018;**17**:128–53. doi:https://doi.org/10.1016/j.gfs.2018.01.002

14 Kadiyala S, Prost A, Harris-Fry H, *et al.* Upscaling Participatory Action and Videos for Agriculture and Nutrition (UPAVAN) trial comparing three variants of a nutrition-sensitive agricultural extension intervention to improve maternal and child nutritional outcomes in rural Odisha, India: study p. *Trials* 2018;**19**:176. doi:10.1186/s13063-018-2521-y

15 Pingali P, Sunder N. Transitioning Toward Nutrition-Sensitive Food Systems in Developing Countries. *Annu Rev Resour Econ* 2017;**9**:439–59. doi:10.1146/annurev-resource-100516-053552

16 Ruel MT, Alderman H. Nutrition-sensitive interventions and programmes: how can they help to accelerate progress in improving maternal and child nutrition? *Lancet* 2013;**382**:536–51. doi:10.1016/S0140-6736(13)60843-0

17 Heidkamp RA, Piwoz E, Gillespie S, *et al.* Mobilising evidence, data, and resources to achieve global maternal and child undernutrition targets and the Sustainable Development Goals: an agenda for action. *Lancet* 2021;**397**:1400–18. doi:10.1016/S0140-6736(21)00568-7

18 Stifel D, Minten B. Market Access, Well-being, and Nutrition: Evidence from Ethiopia. *World Dev* 2017;**90**:229–41. doi:https://doi.org/10.1016/j.worlddev.2016.09.009

19 Ogutu SO, Qaim M. Commercialization of the small farm sector and multidimensional poverty. *World Dev* 2019;**114**:281–93. doi:https://doi.org/10.1016/j.worlddev.2018.10.012

20 Maestre M, Poole N, Henson S. Assessing food value chain pathways, linkages and impacts for better nutrition of vulnerable groups. *Food Policy* 2017;**68**:31–9. doi:https://doi.org/10.1016/j.foodpol.2016.12.007

21 Cooper GS, Shankar B, Rich KM, *et al.* Can fruit and vegetable aggregation systems better balance improved producer livelihoods with more equitable distribution? *World Dev* 2021;**148**:105678. doi:https://doi.org/10.1016/j.worlddev.2021.105678

22 Rammelt CF, Leung MWH. Tracing the Causal Loops Through Local Perceptions of Rural Road Impacts in Ethiopia. *World Dev* 2017;**95**:1–14. doi:https://doi.org/10.1016/j.worlddev.2017.02.024

23 Turner C, Kalamatianou S, Drewnowski A, *et al.* Food Environment Research in Low- and Middle-Income Countries: A Systematic Scoping Review. *Adv Nutr* 2020;**11**:387–97. doi:10.1093/advances/nmz031

24 Turner C, Aggarwal A, Walls H, *et al.* Concepts and critical perspectives for food environment research: A global framework with implications for action in low- and middle-income countries. *Glob Food Sec* 2018;**18**:93–101. doi:https://doi.org/10.1016/j.gfs.2018.08.003

25 Beatty PC, Willis GB. Research Synthesis: The Practice of Cognitive Interviewing. *Public Opin Q* 2007;**71**:287–311. doi:10.1093/poq/nfm006

26 Ashok S, Kim SS, Heidkamp RA, *et al.* Using cognitive interviewing to bridge the intent-interpretation gap for nutrition coverage survey questions in India. *Matern Child Nutr* 2021;**n/a**:e13248. doi:https://doi.org/10.1111/mcn.13248

27 Scott K, Gharai D, Sharma M, *et al.* Yes, no, maybe so: the importance of cognitive

interviewing to enhance structured surveys on respectful maternity care in northern India. *Health Policy Plan* 2020;**35**:67–77. doi:10.1093/heapol/czz141

28 Knight F, Ferguson E. Agrifood; a new Multi-Criteria Decision Analysis tool to examine trade-offs in programme decisions for nutrition sensitive agriculture. In: *Agriculture, Nutrition and Health Academy Week*. Hyderabad, India: 2019. 18.https://www.anh-academy.org/sites/default/files/7A_Frances

29 Gibson R, Ferguson E. An interactive 24-hour recall for assessing the adequacy of iron and zinc intakes in developing countries. Washington DC, U.S.A: 2008. doi:ISBN: 978-0-9818176-1-3

30 Gibson RS, Charrondiere UR, Bell W. Measurement Errors in Dietary Assessment Using Self-Reported 24-Hour Recalls in Low-Income Countries and Strategies for Their Prevention. *Adv Nutr* 2017;**8**:980–91. doi:10.3945/an.117.016980

31 Aslam H Bin, Alarcon P, Yaqub T, *et al.* A Value Chain Approach to Characterize the Chicken Sub-sector in Pakistan. Front. Vet. Sci. 2020;**7**:361.https://www.frontiersin.org/article/10.3389/fvets.2020.00361

32 Alarcon P, Fèvre EM, Murungi MK, *et al.* Mapping of beef, sheep and goat food systems in Nairobi — A framework for policy making and the identification of structural vulnerabilities and deficiencies. *Agric Syst* 2017;**152**:1–17. doi:https://doi.org/10.1016/j.agsy.2016.12.005

33 Deaton A, Cartwright N. Understanding and misunderstanding randomized controlled trials. *Soc Sci Med* 2018;**210**:2–21. doi:https://doi.org/10.1016/j.socscimed.2017.12.005

34 Rich KM, Berends J, Cooper GS. Enriching value chains through maps: reflections from spatial group model building in Myanmar and India. *Dev Pract* 2021;:1–7. doi:10.1080/09614524.2021.1907545

35 Tako AA, Robinson S. Model development in discrete-event simulation and system dynamics: An empirical study of expert modellers. *Eur J Oper Res* 2010;**207**:784–94. doi:10.1016/j.ejor.2010.05.011

36 Cooper GS, Rich KM, Shankar B, *et al.* Identifying 'win-win-win'' futures from inequitable value chain trade-offs: A system dynamics approach'. *Agric Syst* 2021;**190**. doi:10.1016/j.agsy.2021.103096

37 Spear RC, Hornberger GM. Eutrophication in Peel Inlet-II. Identification of critical uncertainties via generalized sensitivity analysis. *Water Res* 1980;**14**:43–9. doi:https://doi.org/10.1016/0043-1354(80)90040-8

Authors contributions:

Gregory S Cooper: Design – development of methods; Writing – led original draft, editing, final version, revisions

Hilary Davies-Kershaw: Design – development of methods; Writing – editing, final version. revisions

Paula Dominguez-Salas: Design – study conceptualisation, development of methods; Writing – contribution to original draft, editing, final version, revisions

Umi Fahmida: Design – study conceptualisation, development of methods; Writing – contribution to original draft, editing

Babacar Faye: Design – study conceptualisation; Writing – editing

Elaine L Ferguson: Design – study conceptualisation, development of methods; Writing – contribution to original draft, editing, final version

Delia Grace: Design – development of methods; Writing – editing

Barbara N Häsler: Design – study conceptualisation, development of methods; Writing – contribution to original draft, editing, final version, revisions

Suneetha Kadiyala: Design – study conceptualisation, development of methods; Writing – contribution to original draft, editing, final version

Archana Konapur: Design – development of methods; Writing – editing, revisions

Bharati Kulkarni: Design – study conceptualisation, development of methods; Writing – contribution to original draft, editing

Bhagyalakshmi C Prakashbabu: Design – development of methods; Writing – editing

Indriya L Pramesthi: Design – development of methods; Writing – editing

Confidential: For Review Only

Dominic Rowland: Design – study conceptualisation, development of methods; Writing – contribution to original draft, editing

Kirtuhika Selvaraj: Design – development of methods; Writing – contribution to original draft, editing

Bhavani Shankar: Design – study conceptualisation, development of methods; Writing – contribution to original draft, editing, final version

Arienta R P Sudibya: Design – development of methods; Writing – editing

Roger C Tine: Design – study conceptualisation, development of methods; Writing – editing

Dinesh Yadav: Design – study conceptualisation, development of methods; Writing – editing

Nur L Zahra: Design – development of methods; Writing – contribution to original draft, editing

Claire Heffernan – Design – study conceptualisation

Funding: This study was funded by UK Research and Innovation Global Challenges Research Fund (UKRI-GCRF) (Project ref. MR/S01313X/1). The views expressed in this work are those of the authors and not the funder. We would also like to express our gratitude to Modou Jobarteh and Kaitlin Conway for their constructive comments on an earlier version of this manuscript.

Conflict of interest statement: The authors declare no conflict of interest.

Confidential: For Review Only

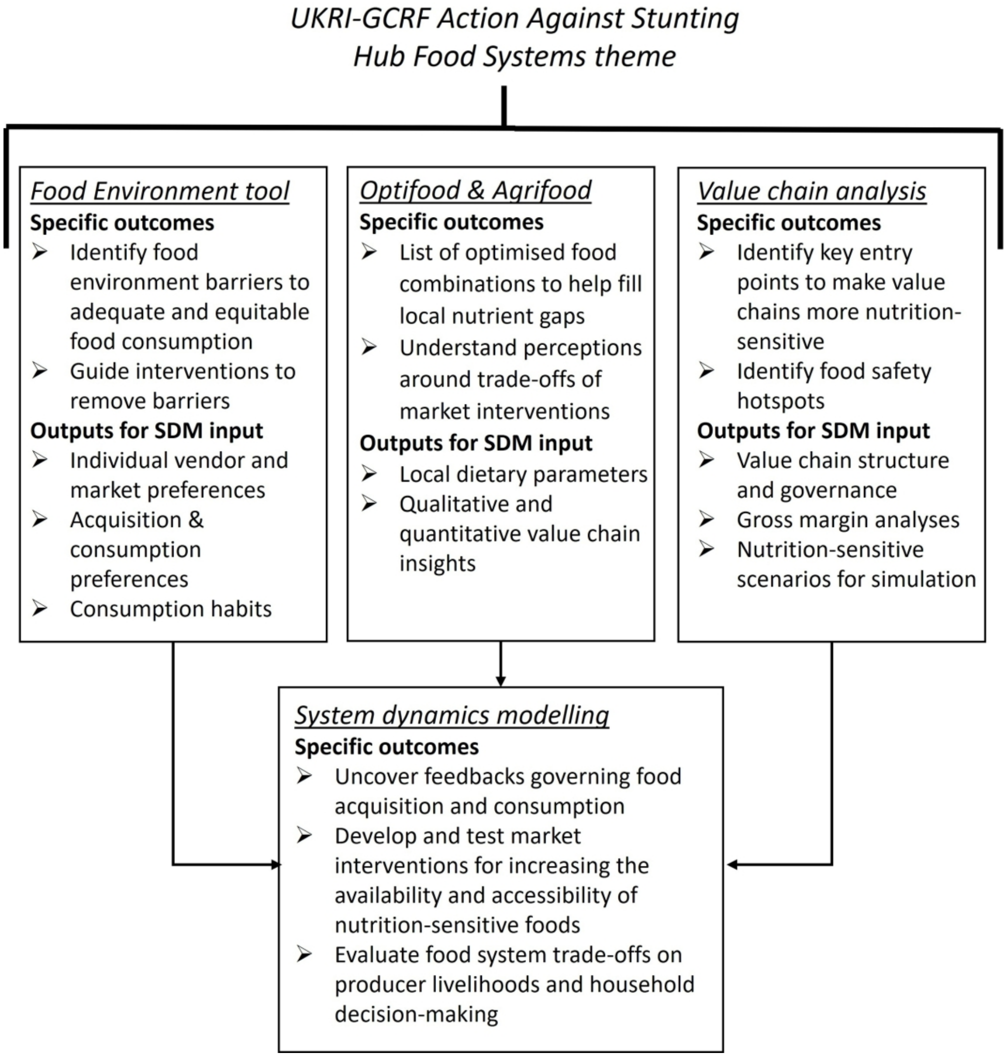

199x212mm (300 x 300 DPI)
